# The siderophore-iron transporter BbMirB is required for the fungal pathogen *Beauveria bassiana* to repress insect immunity and promote proliferation during colonization of hemocoel

**DOI:** 10.1128/mbio.01981-25

**Published:** 2025-08-25

**Authors:** Chenhua Zhu, Qi Liu, Yuhan Chen, Fangfang Tian, Dekun Kong, Isidor Happacher, Hubertus Haas, Yongjun Zhang, Zhibing Luo

**Affiliations:** 1 Key Laboratory of Agricultural Biosafety and Green Production of Upper Yangtze River (Ministry of Education), College of Plant Protection, Southwest University 597769, Chongqing, China; 2 College of Pharmaceutical Sciences, Southwest University 26463, Chongqing, China; 3 Institute of Molecular Biology, Biocenter, Medical University of Innsbruck 27280, Innsbruck, Austria; 4 Key Laboratory of Entomology and Pest Control Engineering, Academy of Agricultural Sciences, Southwest University 630662 , Chongqing, China; Universidade de Sao Paulo, Ribeirao Preto, Sao Paulo, Brazil

**Keywords:** insect pathogenic fungus, nutritional immunity, siderophore-iron transporter, proliferation, virulence

## Abstract

Nutritional immunity plays a vital role in limiting the proliferation of microbial pathogens in vertebrates and invertebrates. During coevolution with their hosts, pathogens have developed tactics to counteract the host nutritional immunity by acquiring iron via siderophore-iron transporter (SIT)-mediated uptake of siderophore-iron chelates. However, little is known about the role of SITs of insect fungal pathogens in combating host immune defense. Here, we demonstrate that *Beauveria bassiana*, an important insect pathogenic fungus, modulates dominant extracellular siderophores and co-expresses the SITs *BbMirA* and *BbMirB* for adaptation to the host *in vivo*. BbMirB, but not BbMirA, is crucial for *B. bassiana* full virulence. Disruption of *BbMirB* significantly reduces the virulence against *Galleria mellonella* larvae, delays immune evasion and *in vivo* hyphal body proliferation, and alters the host immune responses, including host nutritional immunity and immune-related gene expression. Loss of BbMirA or BbMirB leads to increased extracellular coprogen-type siderophores, including coprogen B, dimerumic acid, and their derivatives, suggesting these metabolites as their potential substrates. BbMirA and BbMirB co-localized to the plasma membrane, early/late endosomes, and vacuoles, implicating their roles in vesicular trafficking and siderophore-iron uptake. These findings highlight the importance of siderophore-iron transporters, particularly BbMirB, in fungal vesicular trafficking and host-pathogen interactions.

**IMPORTANCE:**

Siderophores are essential for iron uptake under iron-limiting conditions and are involved in fungal niche competition and pathogenicity via siderophore-iron transporters (SITs)-mediated uptake of specific substrates. However, the details of many SIT-mediated substrates in fungal colonization of hosts remain limited. Here, we identify two SITs, BbMirA and BbMirB, in the entomopathogenic fungus *Beauveria bassiana*, which are highly expressed during colonization of insect hemocoel. The two SITs have complementary functions in siderophore-iron uptake *in vitro*, but only BbMirB dominantly mediates the uptake of a derivative of dimerumic acid *in vivo* and plays a critical role in the pathogenic process via disturbing insect immune defense responses. These findings provide insights into the mechanisms of SITs mediating the interaction of fungal pathogens with their hosts.

## INTRODUCTION

Iron is essential for all organisms, supporting respiration, DNA synthesis, and cell division. However, iron availability is often limited in biological environments, making its acquisition vital for survival (1, 2). Both vertebrates and invertebrates employ iron sequestration as a form of nutritional immunity to restrict pathogen proliferation *in vivo* (3, 4). In response, fungal pathogens have evolved high-affinity siderophore-mediated iron acquisition (SIA) to thrive in iron-deficient environments (1, 5).

Siderophores are low-molecular-weight metabolites produced by bacteria and fungi under iron-limited conditions, which can tightly chelate ferric iron forming siderophore-iron complexes. Most fungal siderophores belong to the hydroxamate type, with biosynthesis initiated by L-ornithine-N^5^-hydroxy-monooxygenase (SidA) converting L-ornithine to N^5^-hydroxyornithine (5). SidA inactivation blocks production of these siderophores, increasing iron starvation sensitivity and attenuating virulence across diverse fungal pathogens, including the human pathogen *Aspergillus fumigatus* (6, 7), the plant pathogens *Magnaporthe grisea* (8) and *Fusarium graminearum* (9), and the entomopathogenic fungi *Nomuraea rileyi* (10) and *Beauveria bassiana* (11).

Fungal siderophores comprise fusarinines, coprogens, ferrichromes, and rhodotorulic acid, including various O-glycosylated and N-oxidized derivatives (5, 12). The link between siderophore biosynthesis and virulence has been extensively explored by disrupting core genes within siderophore biosynthesis clusters apart from SidA. For example, inactivation of the nonribosomal peptide synthetase NPS6 blocks the production of triacetylfusarinine C (TAFC) and coprogen-type siderophores, leading to reduced virulence in plant pathogenic ascomycetes (13–15). Similar results have been observed with mutants lacking homologous gene in animal pathogenic *A. fumigatus* (6) and *Scedosporium apiospermum* (16), as well as plant pathogenic *M. grisea* (8). However, in some cases, siderophore biosynthesis appears to have species-specific contributions to virulence. For instance, blocking biosynthesis of coprogen-type siderophore(s) via inactivation of sidD attenuates virulence in *B. bassiana* ARSEF2860 (11), but not in *Metarhizium robertsii* (17). Similarly, abrogation of biosynthesis of ferrichrome-type ferricrocin by *sidC*/*ferS* deletion in *B. bassiana* reduces the virulence of strain ARSEF2860 (11) but increases the virulence of strain BCC2660 (18). These findings highlight the functional diversity of siderophores and their differential roles in fungal virulence across species and strains. With the current knowledge, it is difficult to selectively block the synthesis of individual siderophores, complicating the assessment of their specific contributions to specific niches. For example, inactivation of either *sidD* or *sidF* or *sidI* blocks biosynthesis of TAFC and FsC production in *A. fumigatus* (6) and production of six coprogen- and fusarinine-type siderophores in *B. bassiana* (11).

Siderophore-iron complexes enter fungal cells via siderophore-iron transporters (SITs), which exhibit varying substrate specificities (19). Most fungi possess multiple SITs for utilizing both endogenous siderophores and xenosiderophores, a crucial adaptation for ecological versatility. Even non-siderophore-producing *Saccharomyces cerevisiae* has four siderophore transporters (5). In *A. fumigatus*, four SITs have been characterized: MirB and MirD transport TAFC and fusarinine C (20), respectively, while Sit1 and Sit2 transport ferrichrome- and coprogen-type siderophores, with Sit1 additionally transporting ferrioxamine-type siderophores (21). Only MirB is essential for *A. fumigatus* virulence in murine models (20). These results illustrate that specific transporters are likely to play niche-specific roles in iron uptake but do not uniformly contribute to virulence. SITs belong to the major facilitator superfamily, typically containing 12–14 transmembrane domains. While certain SIT substrates have been identified through heterologous expression systems and radioisotope-labeled siderophores (20, 22), the precise mechanisms of substrate transport remain debated: direct transmembrane translocation (23, 24) or endocytosis-mediated uptake (25).

*B. bassiana* is a versatile biocontrol fungus that infects insects and colonizes plants, promoting growth and enhancing resistance to pathogens and pests (26). More than 15 typical siderophores have been identified in *B. bassiana*, suggesting that their functions may extend beyond iron acquisition (11, 18, 27). The *B. bassiana* genome encodes various numbers of SITs across different strains (28, 29), providing alternative mechanisms for competing for iron in diverse environments. However, the functional associations between specific SITs and siderophores, and their ecological roles, remain unclear. Understanding the roles of SITs in nutritional competition between fungal pathogens and their hosts may provide novel insights into fungal pathogenic mechanisms.

Here, we investigated two SITs, BbMirA and BbMirB, which are highly expressed during the insect-colonizing stage in *B. bassiana*. Our findings identify a derivative of dimerumic acid (formula C_22_H_40_N_4_O_10_), primarily transported by BbMirB and *trans*-fusarinine as the predominant siderophores within *B. bassiana*-infected insects. Both transporters facilitate the translocation of siderophore-iron complexes from the extracellular environment to vacuoles via vesicular trafficking. Notably, BbMirB demonstrates a more significant role in immune evasion and fungal proliferation within the host, underscoring the collaborative function of these transporters in modulating host-pathogen interactions.

## RESULTS

### Identification of *SITs* that are highly expressed during *B. bassiana* colonization of the insect hemocoel

**Fig 1:**
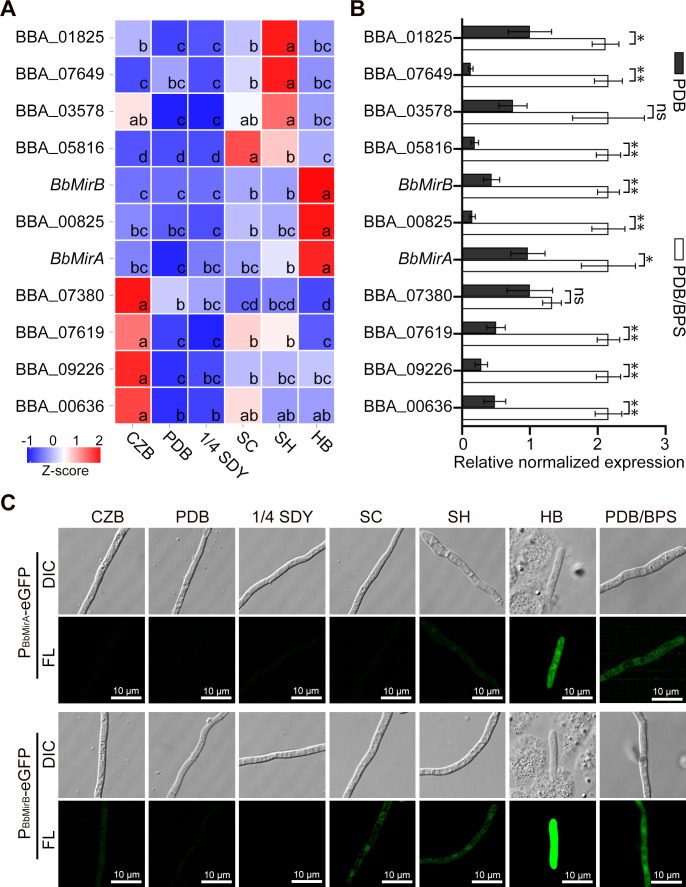
Expression patterns of SIT-encoding genes in *B. bassiana*. (**A**) RT-qPCR analysis of *SITs* in wild type (WT) grown in various nutrient conditions or insect hemocoel using 18S rRNA gene as a reference. Normalized fold expression values (2^−ΔΔ*C*t^, mean ± SE, *n* = 3) were rescaled (*z*-score algorithm) and plotted on the heatmap graphic. Genes are clustered along the y-axis, and the scale bar indicates colors corresponding to each *z*-score in the expression heatmap. “Zero” denotes the mean *z*-score value of the x-axis distribution. Lack of identical lowercase letters indicates statistically significant differences (*P* < 0.05, least significant difference (LSD) or Dunnett’s T3 test). (**B**) RT-qPCR analysis of *SITs* in WT under normal (PDB) or iron-limited (PDB/bathophenanthroline disulfonic acid [BPS]) conditions (mean ± SE, *n* = 3). ** and * indicate significant differences at *P* < 0.01 and *P* < 0.05, respectively, using two-tailed Student’s *t*-test. ns, no significant difference (*P* > 0.05). (**C**) Fluorescence microscopy of *P_BbMirA_-eGFP* or *P_BbMirB_-eGFP*. CZB, Czapek-Dox broth. PDB, potato dextrose broth. 1/4SDY, diluted 1:4 of Sabouraud dextrose broth amended with 1% yeast extract. SC and SH, the basic salt broth supplemented with 1.67 g/L silkworm cuticle or 5 mL/L hemolymph. HB, *in vivo* hyphal bodies. PDB/BPS, PDB supplemented with 200 µM BPS. Bar = 10 µm.

To identify highly expressed *SIT*s during fungal colonization of the host hemocoel, their expression profiles were determined in fungal cells grown under various nutrient conditions or within insects, using the reverse transcription real-time quantitative PCR (RT-qPCR). Only 3 out of 11 putative *SITs* (BBA_00825, BBA_08473, and BBA_05827) exhibited high transcript levels in blastospores grown within insects (also known as *in vivo* hyphal bodies), while most *SITs*, except for BBA_07380 and BBA_03578, were significantly upregulated under iron-limited conditions (Fig. 1A and B). Conserved domain analysis revealed that all 11 putative SITs in *B. bassiana*, except for BBA_00825 with PF06609, share the PF07690 (Fig. S1A through C). In this work, we focused on BBA_08473 and BBA_05827, here termed BbMirA and BbMirB, respectively. To validate the expression patterns, strains carrying enhanced green fluorescence protein (eGFP)-tagged versions, cassettes *P_BbMirA_-eGFP* and *P_BbMirB_-eGFP*, respectively, were analyzed using confocal laser scanning microscopy. Strong fluorescence signals were examined in fungal cells grown in either the host hemocoel or iron-limited broth (Fig. 1C), confirming the RT-qPCR results.

Fungal siderophore biosynthetic genes tend to be clustered in the genome (5). *In vivo* transcriptomic data from *B. bassiana* revealed that of the three (putative) siderophore biosynthetic clusters, only one (BBA_06993–BBA_06997) was highly expressed (30), which is consistent with our examination using RT-qPCR (Fig. S1D and E), exhibiting co-expression patterns with the transporters BbMirA and BbMirB. A recent study has demonstrated that the cluster (BBA_06993–BBA_06997) is responsible for coprogen-type siderophore biosynthesis in *B. bassiana* ARESF 2860 strain (11). These results suggest that BbMirA and BbMirB may be involved in the transport of coprogen-type siderophores.

### BbMirB, but not BbMirA, is crucial for full virulence of *B. bassiana*

**Fig 2:**
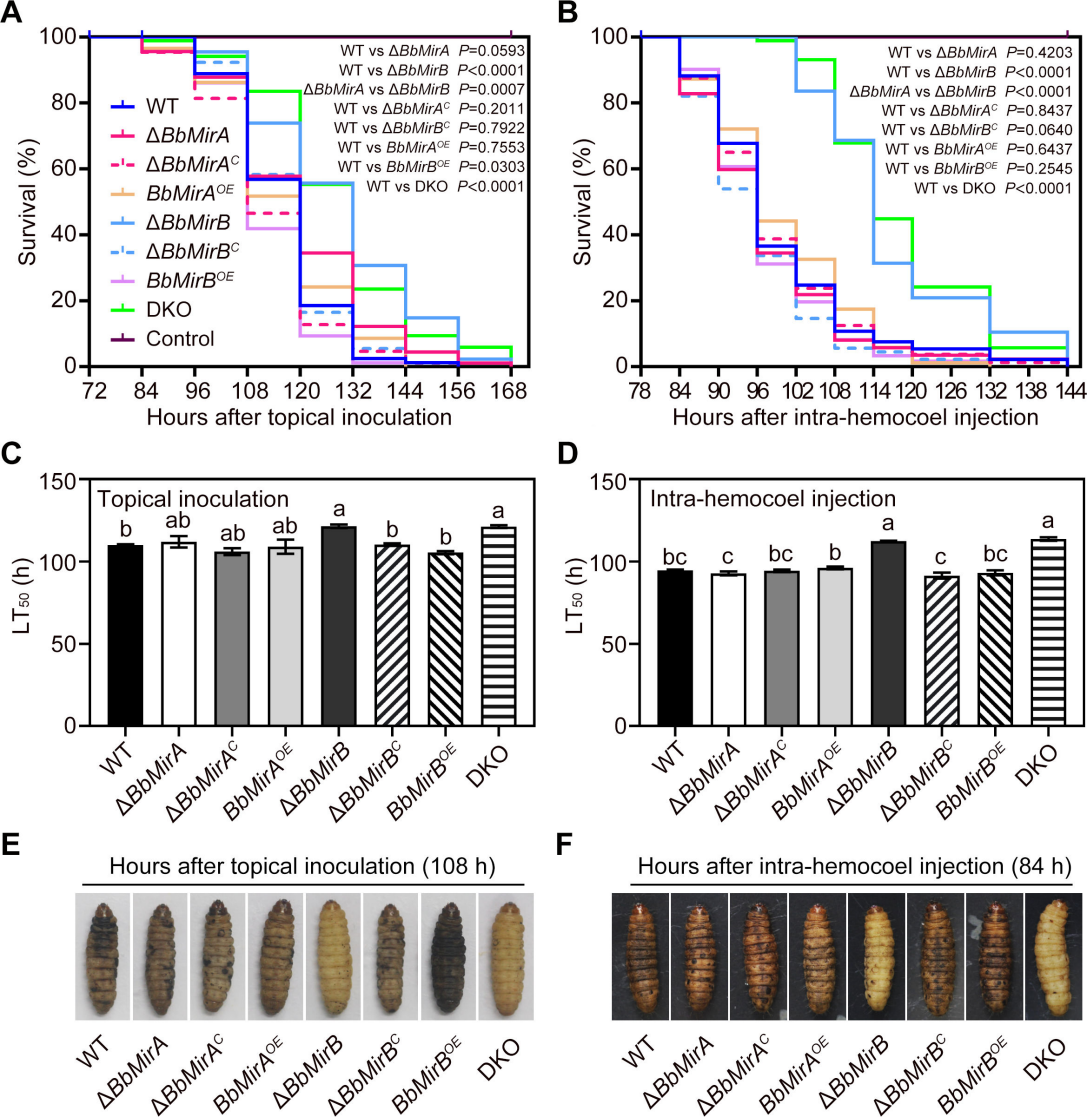
Insect bioassays. (**A**) Insect survival after topical inoculation. (**B**) Insect survival after intra-hemocoel injection. (**C**) The LT_50_ values of topical bioassays (mean ± SE, *n* = 3). (**D**) The LT_50_ values of injection bioassays. (**E and F**) Symptoms of *G. mellonella* larvae via topical infection (**E**) and intra-hemocoel injection (**F**) at the indicated time. Statistical significance was assessed via a Log-rank test for pairwise survival curve comparisons at *P* < 0.05 (**A and B**). Lack of identical lowercase letters indicates statistically significant differences between groups (*P* < 0.05, Dunnett’s T3 or least significant difference [LSD] test) (**C and D**).

To assess the roles of BbMirA and BbMirB in virulence of *B. bassiana*, single- and double-knockout mutants (Δ*BbMirA*, Δ*BbMirB*, and DKO), the complemented (Δ*BbMirA^C^* and Δ*BbMirB^C^*), and overexpressing (*BbMirA^OE^* and *BbMirB^OE^*) strains were generated as described in the section “Materials and Methods” (Fig. S2). Bioassays using last instar larvae of *Galleria mellonella* via topical infection revealed that knockout of *BbMirB* or the double knockout of *BbMirA* and *BbMirB* (DKO) significantly reduced the virulence of *B. bassiana* (*χ^2^* = 28.21, *P* < 0.0001 or *χ^2^* = 29.73, *P* < 0.0001; Fig. 2A). The median lethal time (LT_50_) values for Δ*BbMirB* and DKO were 121.4 ± 1.5 hours and 121.2 ± 1.2 hours, respectively, an increase of 10.4% and 10.2% compared to the wild type (WT; 110.0 ± 0.8 hours; Fig. 2C). Similarly, in intra-hemocoel injection assays, both Δ*BbMirB* and DKO showed compromised virulence (*χ^2^* = 54.69, *P* < 0.0001 or *χ^2^* = 71.18, *P* < 0.0001; Fig. 2B), with significantly higher LT_50_ values (112.5 ± 0.3 and 113.7 ± 1.5 hours) compared to the WT (94.7 ± 0.8 hours; Fig. 2D). No obvious difference in virulence was examined between Δ*BbMirB^C^* and WT strains. By contrast, overexpression of *BbMirB* slightly increased virulence in topical bioassays but caused no change in intra-hemocoel injection assays (Fig. 2A and B). In contrast, disruption of *BbMirA* in the WT or Δ*BbMirB*, or overexpression of *BbMirA*, did not affect virulence as compared with their parent strains (WT and Δ*BbMirB*; Fig. 2A through D), suggesting no significant contribution of BbMirA to the fungal pathogenicity. Notably, larvae infected with Δ*BbMirB* or DKO through either topical application or intra-hemocoel injection exhibited lighter melanization compared to insects infected with the other strains (Fig. 2E and F), indicating a potential role of BbMirB in suppressing/evading host immune response.

### Disruption of *BbMirB* retarded fungal immune evasion and proliferation within the insect hemocoel

**Fig 3:**
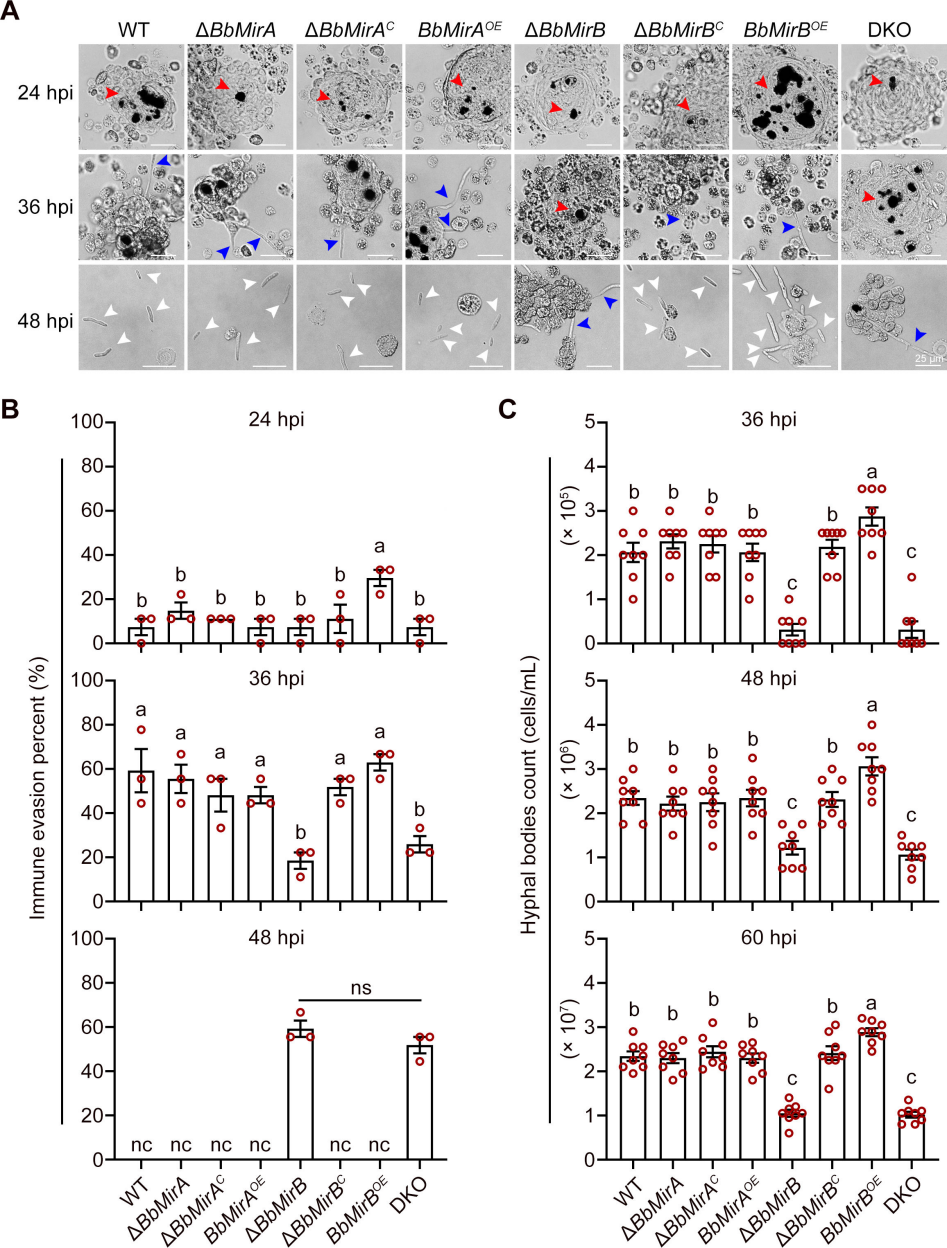
Fungal immune evasion and proliferation *in vivo*. (**A**) Microscopic images of fungal development *in vivo*. Red, blue, and white arrows indicate melanized nodules (hemocyte encapsulation), evading hyphae, and free hyphal bodies *in vivo*, respectively. Bar = 25 µm. (**B**) Fungal immune evasion. Total of evading and encapsulated events was counted at the indicated time point, and evasion ratio was calculated (mean ± SE, *n* = 3). Lack of identical lowercase letters between groups indicates statistically significant differences (*P* < 0.05, least significant difference [LSD] test). nc, not calculated due to no observed encapsulated but all free hyphal bodies. ns, no significance (*P* > 0.05, Student’s *t*-test). (**C**) Counts of *in vivo* hyphal bodies (mean ± SE, *n* = 8). Lack of identical lowercase letters between groups indicates statistically significant differences (*P* < 0.05, least significant difference [LSD] test).

*G. mellonella* larvae were subjected to intra-hemocoel injections, and subsequent formation of hemocyte encapsulation, melanized nodules, and immune evasion was monitored at 24, 36, and 48 hours post-infection (hpi). These analyses revealed germinating conidia surrounded by hemocytes and the germ tube penetration through the encapsulation, leading to free *in vivo* hyphal body formation (Fig. 3A). The *BbMirB* overexpression strain demonstrated enhanced immune evasion, with 29.6% of germ tubes penetrated through hemocyte encapsulation at 24 hpi compared to wild type (7.4%; Fig. 3B). Conversely, both Δ*BbMirB* and DKO exhibited significantly reduced capabilities of immune evasion at 36 hpi (18.5% and 25.9%, respectively) vs wild type (59.3%). By 48 hpi, 59.3% and 51.9% of germinating conidia from Δ*BbMirB* and DKO remained encapsulated, while no encapsulation but free hyphal bodies *in vivo* were observed in other strains. Furthermore, production of hyphal bodies *in vivo* was significantly delayed in Δ*BbMirB* and DKO mutants compared to WT, with respective reductions of 84.8% for both mutants at 36 hpi, 48.0% and 54.7% at 48 hpi, and 55.2% and 56.5% at 60 hpi, respectively (Fig. 3C). In contrast, the overexpressing strain *BbMirB^OE^* produced a higher number of *in vivo* hyphal bodies, with increases of 39.4%, 30.7%, and 23.2%, respectively. Whereas, disruption or overexpression of *BbMirA* did not affect the fungal proliferation within the host hemocoel.

### Disruption of *BbMirA* and *BbMirB* affects host immune responses differently

**Fig 4:**
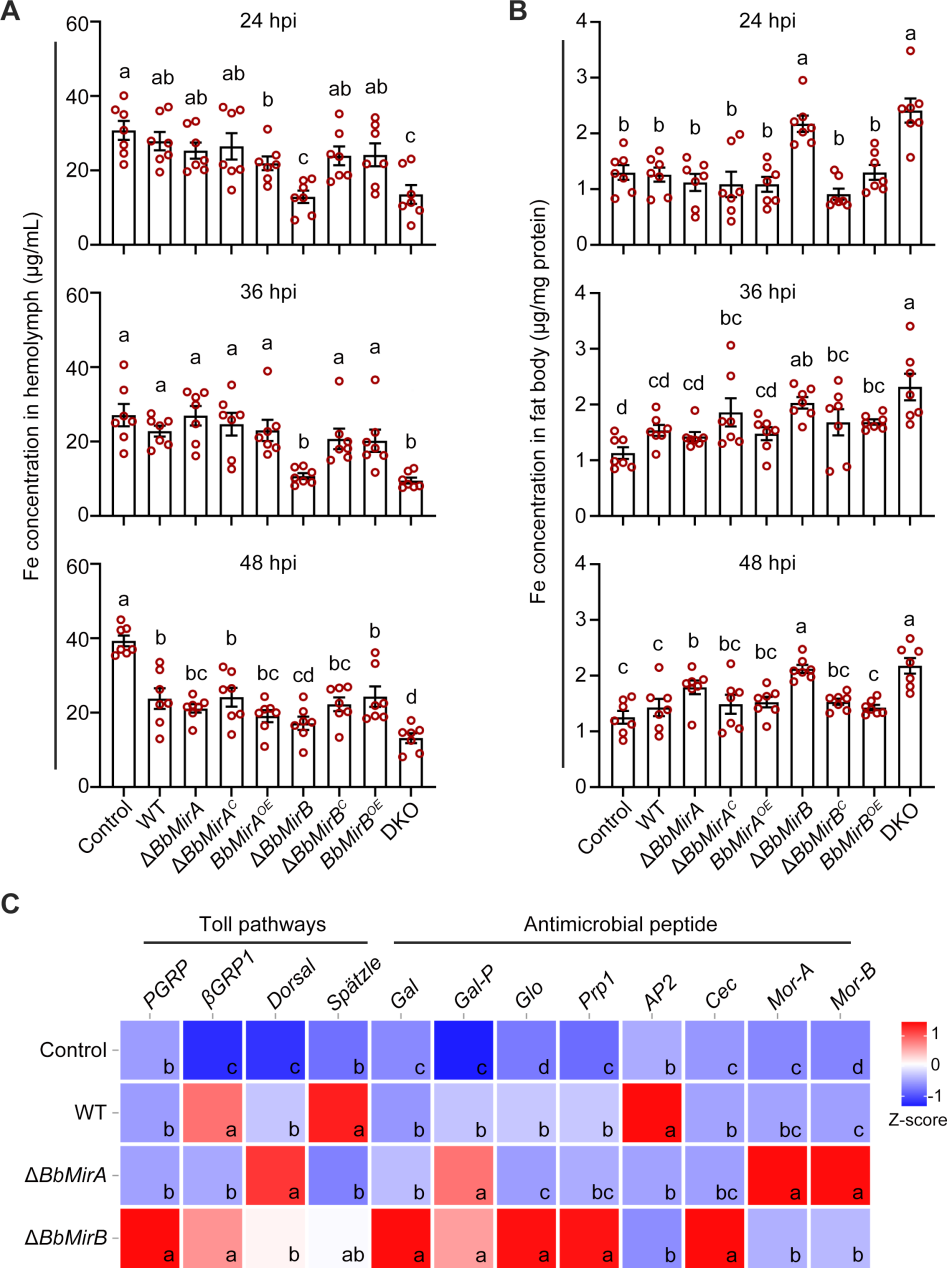
Insect immune responses to fungal colonization. (**A and B**) Total iron content in insect hemolymph (**A**) and fat body (**B**) derived from infected *G. mellonella* larvae via intra-hemocoel injection at the indicated time (mean ± SE, *n* = 7). Control, larvae injected with sterile 0.9% NaCl. Lack of identical lowercase letters between groups indicates statistically significant differences (least significant difference [LSD] test, *P* < 0.05). (**C**) RT-qPCR analysis of immune-related gene expression in fat body using *G. mellonella* β-actin gene as a reference. Normalized fold expression values (2^−ΔΔ*C*t^, mean ± SE, *n* = 3) were rescaled (*z*-score algorithm) and plotted on the heatmap graphic. The scale bar indicates colors corresponding to each *z*-score in the expression heatmap. “Zero” denotes the mean *z*-score value of the x-axis distribution. Lack of identical lowercase letters between groups indicates statistically significant differences (LSD or Dunnett’s T3 test, *P* < 0.05).

To investigate the impact of *BbMirA* and *BbMirB* disruption or overexpression on host nutritional immunity, total iron concentrations were measured in the hemolymph and fat body cells of infected larvae via intra-hemocoel injection. Δ*BbMirB*- or DKO-infected insects exhibited a significantly reduced hemolymph iron content compared to WT-treated group, decreasing by 53.7% and 51.4% at 24 hpi, 53.0% and 58.3% at 36 hpi, and 27.9% and 44.7% at 48 hpi, respectively (Fig. 4A). Conversely, iron concentrations of fat body in Δ*BbMirB* or DKO-infected larvae were significantly elevated compared to the WT-treated group, increasing by 72.3% and 90.9% at 24 hpi, 32.2% and 50.8% at 36 hpi, and 48.4% and 52.3% at 48 hpi, respectively (Fig. 4B). However, larvae infected with other strains showed no significant differences in iron content of either hemolymph or fat body compared to the WT-treated insects (Fig. 4A and B), consistent with the results from bioassays.

To explore changes in insect immune responses, we further examined the expression of key genes in the Toll pathway and antimicrobial peptides (Table S2) in larvae injected with Δ*BbMirA*, Δ*BbMirB*, or WT strain. Compared to control insects infected with wild-type strain, the *PGRP* gene in the Toll signaling pathway and antimicrobial peptide genes *Gal*, *Gal-P*, *Glo*, *Prp1*, *Cec*, and *Mor-B* were significantly upregulated in the fat body of Δ*BbMirB*-infected larvae, with significant downregulation of *AP2* (Fig. 4C). In contrast, Δ*BbMirA*-infected larval fat body exhibited significant upregulation of only *Dorsal*, *Gal-P*, *Mor-A*, and *Mor-B*, with significant downregulation of *βGRP*, *Spätzle*, *Glo*, and *AP2*, while expression levels of other examined immune-related genes showed no significant differences.

### Contributions of BbMirA and BbMirB to multistress tolerance

**Fig 5:**
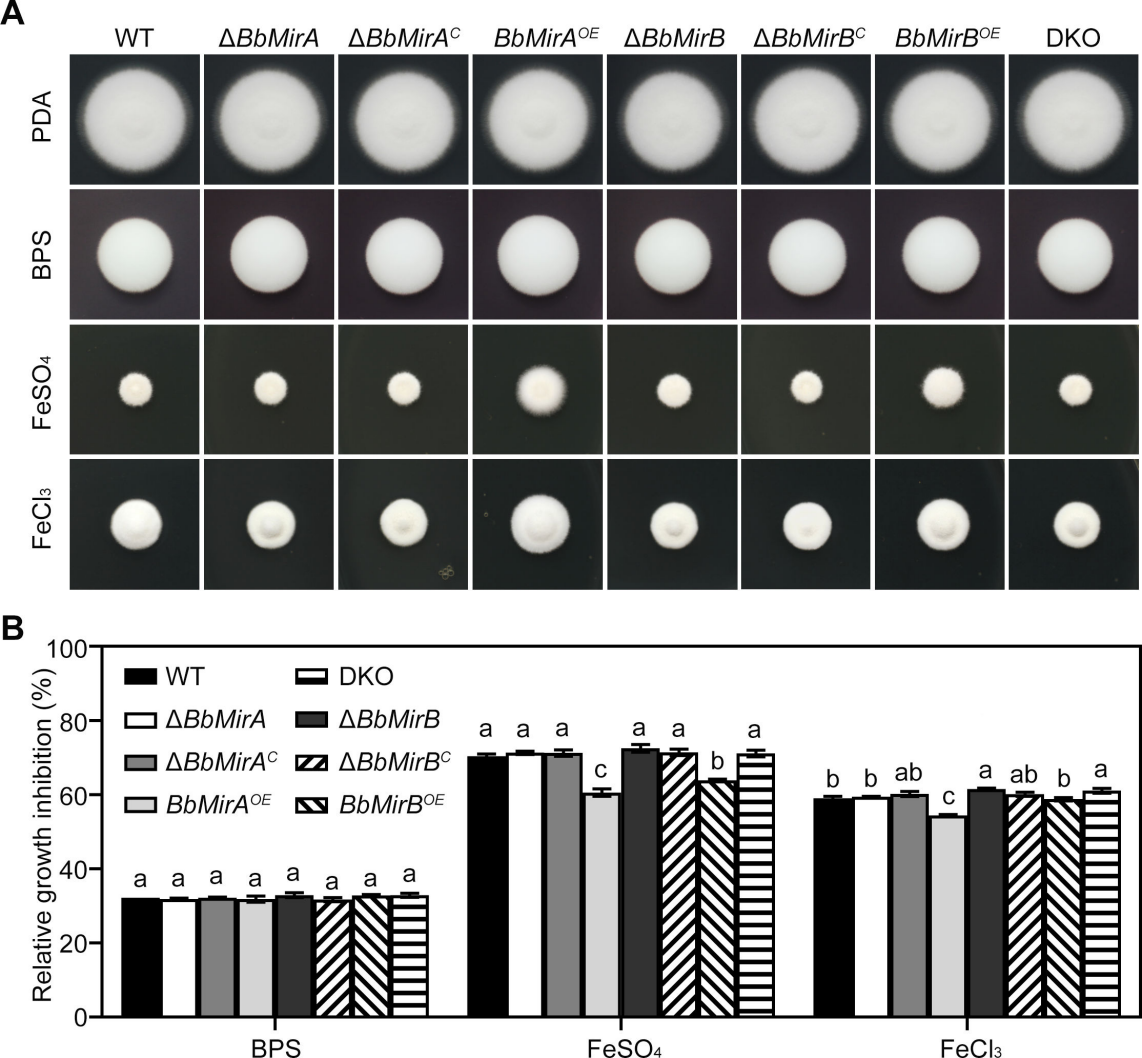
Growth phenotypes under iron-stressed conditions. (**A**) Fungal growth under iron-limited or replete stresses. Equivalent conidial suspensions (2 µL 10^7^ conidia/mL) were applied on PDA, or PDA with 200 µM BPS for iron-starvation, 7.5 mM FeSO_4_ or 2.5 mM FeCl_3_ for iron-replete stresses, respectively, and incubated at 26°C for 7 days. (**B**) Values of relative growth inhibition were calculated according to the colony diameters (mean ± SE, *n* = 3). Lack of identical lowercase letters between groups indicates statistically significant differences (*P* < 0.05, least significant difference [LSD] test).

To assess BbMirA and BbMirB contributions to fungal iron homeostasis, growth of WT and genetically modified strains was evaluated under iron-limited or iron-replete conditions (Fig. 5). All tested strains exhibited comparable growth on PDA or PDA with 200 µM bathophenanthroline disulfonic acid (BPS), suggesting that *BbMirA* and/or *BbMirB* disruption did not affect fungal tolerance to iron starvation. Conversely, *BbMirA^OE^* and *BbMirB^OE^* strains demonstrated enhanced tolerance to Fe^2+^ stress, reducing FeSO_4_-induced relative growth inhibition values by 13.9% and 9.2%, respectively. Under Fe^3+^ stress, only *BbMirA^OE^* exhibited significant protection (7.9% reduction of relative growth inhibition values), while *BbMirB^OE^* showed no appreciable effect. Notably, Δ*BbMirB* and DKO mutants displayed slightly increased Fe³^+^ sensitivity, with decrements of 4.2% and 3.5% in relative growth inhibition values, respectively. Additionally, Δ*BbMirA* and DKO, but not Δ*BbMirB*, exhibited increased tolerance to hyperosmotic stress (1.4 M sorbitol) compared to the WT, showing reduced relative growth inhibition by 23.4% and 17.6%, respectively (Fig. S3A and B). However, no differences were observed among all tested strains under salt (1.3 M NaCl or 1.5 M KCl) stress. In response to oxidative stress (H₂O₂), Δ*BbMirA* and DKO mutants showed increased relative growth inhibition values by 12.6% and 11.1%, respectively, compared to the WT (Fig. S3C and D). Overexpression of *BbMirA* slightly enhanced fungal tolerance to H_2_O_2_ and tert-butyl hydroperoxide (t-BHP), with decreased relative growth inhibition values by 11.3% and 26.2%, respectively. In contrast, only *BbMirB^OE^* showed a minor increase in tolerance to the oxidant menadione (MND) with a decline in relative growth inhibition value by 13.0%.

### Ablation of BbMirA or BbMirB increases the production of extracellular coprogen-type siderophores *in vitro* and *in vivo*

**Fig 6:**
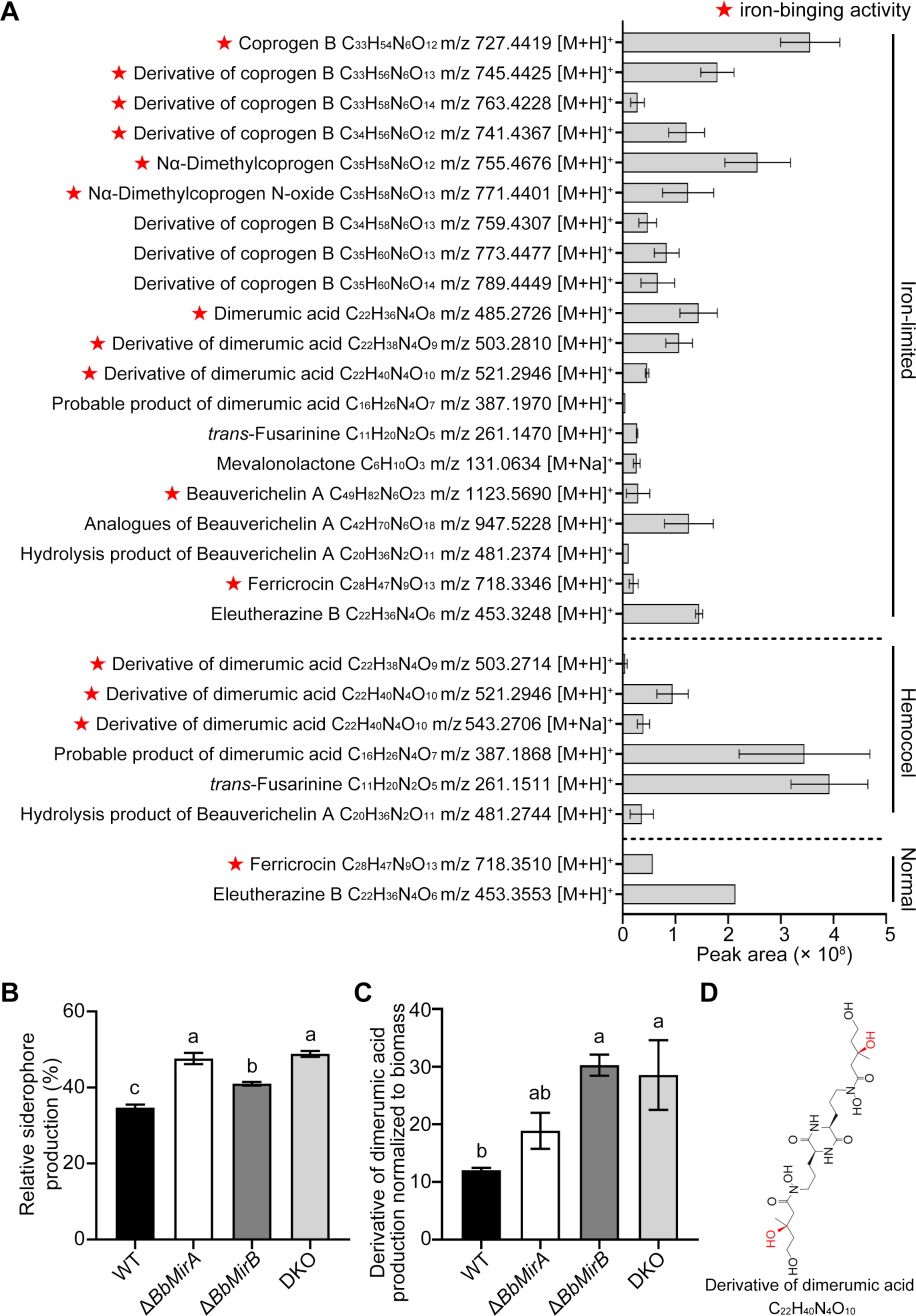
Siderophore identification. (**A**) Siderophores secreted by *B. bassiana* WT grown in PDB, PDB with BPS (iron-depleted), or host hemocoel (*in vivo*; mean ± SE, *n* = 2). ^★^ Represents the siderophores with the potential ability to chelate iron. (**B**) Extracellular siderophores produced by WT, Δ*BbMirA*, Δ*BbMirB*, and DKO, respectively, grown under iron-depleted conditions (mean ± SE, *n* = 3). (**C**) Production (peak area relative to *in vivo* hyphal bodies) of a typical siderophore (a derivative of dimerumic acid) in host hemolymph infected by indicated strains (mean ± SE, *n* = 3). (**D**) The molecular structure of the major derivative of dimerumic acid *in vivo*. Lack of identical lowercase letters between groups indicates statistically significant differences (*P* < 0.05, least significant difference [LSD] test) (**B and C**).

Identification of the secreted siderophores of *B. bassiana* Bb0062 revealed about 20 siderophore-linked metabolites under iron limitation, five within the host hemocoel, and two under normal conditions (Fig. 6A; Fig. S4A), suggesting their distinct roles in fungal adaptation to diverse niches and/or different metabolization. Total extracellular siderophore production was significantly higher in Δ*BbMirA*, Δ*BbMirB*, and DKO strains—increasing by 37.1%, 18.0%, and 40.6%, respectively—compared to the WT, with no significant differences between Δ*BbMirA* and DKO (Fig. 6B). Further identification indicated that Δ*BbMirA* and Δ*BbMirB* show significantly higher amounts of coprogen B and dimerumic acid, along with their derivatives *in vitro*, compared to the WT (Table S3). We also found that the *B. bassiana* Bb0062 mutant lacking the nonribosomal peptide synthetase BbSidD (BBA_06997) exhibits a loss of coprogen-type siderophores, including coprogen B, dimerumic acid, and its derivative (formula C_22_H_38_N_4_O_9_), along with increased ferricrocin production, but unchanged *trans*-fusarinine levels under iron-limited conditions (Fig. S4B).

Since the expression of *BbMirA* and *BbMirB*, along with the coprogen-type siderophore biosynthetic cluster (BBA_06993–BBA_06997), was significantly upregulated in blastospores *in vivo* (30), we analyzed the extracellular siderophores in the hemolymph of larvae infected by each mutant for identification of their potential substrates. Notably, a molecule with an approximate mass of 520.58 Da (m/z 543.36 [M + Na]^+^) was increased in hemolymph infected by Δ*BbMirB* or DKO compared to the WT-treated group, with its relative abundance (based on ratio of peak area to biomass) increasing approximately 2.51- and 2.37-fold, respectively (Fig. 6C). However, no significant differences in the production were observed between Δ*BbMirA* and WT or between Δ*BbMirB* and DKO. According to the molecular mass and a previous report (27), this molecule was assumed to be a derivative (formula C_22_H_40_N_4_O_10_, Fig. 6D) of dimerumic acid, a degradation product of coprogen-type siderophores. In line, Δ*BbsidD* of *B. bassiana* Bb0062 failed to produce this siderophore within the host hemocoel (Fig. S4C). In addition, disruption of *BbSidD* compromised the virulence (*χ*^2^ = 48.88, *P* < 0.0001) and increased the sensitivity to iron starvation (Fig. S4D through F), consistent with a recent report (11).

### Both BbMirA and BbMirB are trafficked in vesicles

**Fig 7:**
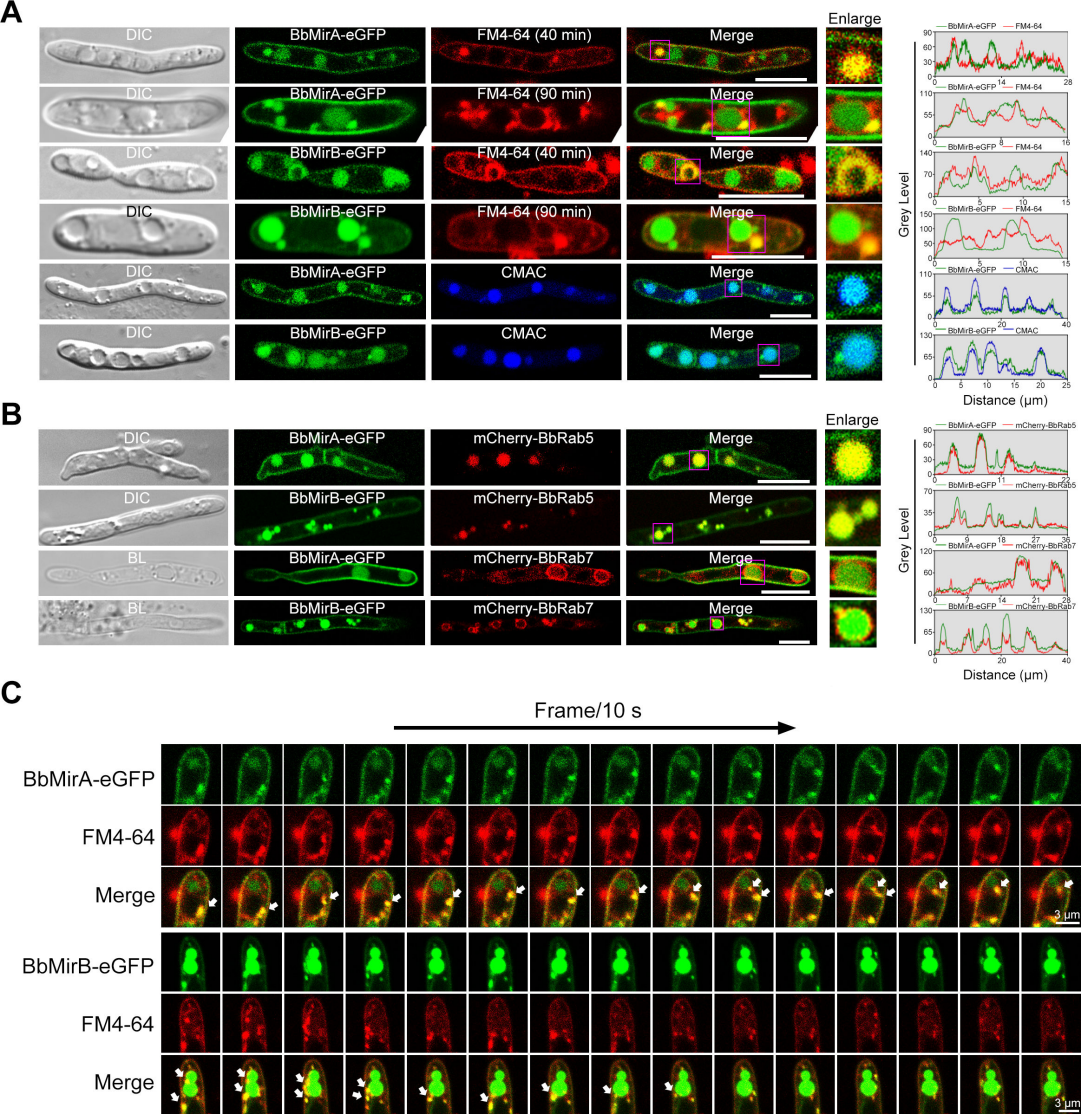
Subcellular localization and trafficking of BbMirA and BbMirB. (**A**) BbMirA and BbMirB localized to the plasma membrane and vacuoles. *In vivo* hyphal bodies of *BbMirA-eGFP* or *BbMirB-eGFP* were isolated from injected larvae at 48 hpi and stained with FM4-64 or CMAC for indicating the plasma membrane or vacuoles, respectively. (**B**) BbMirA and BbMirB localized to the EEs and LEs. *In vivo* hyphal bodies carrying BbMirA-eGFP/mCherry-BbRab5, BbMirB-eGFP/mCherry-BbRab5*,* BbMirA-eGFP/mCherry-BbRab7, or BbMirB-eGFP/mCherry-BbRab7, respectively, were derived from injected larvae at 48 hpi. (**C**) Trafficking of BbMirA and BbMirB. The isolated *in vivo* hyphal bodies of *BbMirA-eGFP* or *BbMirB-eGFP* were stained with FM4-64 for 60 min to examine the target proteins’ trafficking. All treated *in vivo* hyphal bodies were examined using confocal microscopy. Co-localization of the proteins, or protein with dye-stained organelles, was further evaluated by linescan graph analysis using Image J (horizontal axis indicates the distance). White arrows indicated the moving vesicles. Bar = 8 µm (**A and B**) or 3 µm (**C**).

To determine the subcellular localization of BbMirA and BbMirB, BbMirA-eGFP and BbMirB-eGFP fusion proteins were expressed in Δ*BbMirA* and Δ*BbMirB*, respectively. Fluorescence microscopy of *in vivo* hyphal bodies revealed that both BbMirA-eGFP and BbMirB-eGFP were partially localized to the plasma membrane, as indicated by co-localization with the FM4-64 marker, and to vacuoles stained by 7-amino-4-chloromethylcoumarin (CMAC; Fig. 7A). Additionally, partial intracellular signals from the hybrid proteins were observed in globular-like structures, which did not overlap with CMAC, potentially representing the endosomes prior to vacuole fusion (31). The hybrid marker proteins, mCherry-BbRab5 and mCherry-BbRab7, were used to label early endosomes (EEs) and late endosomes (LEs), respectively. Fluorescence microscopy of *in vivo* hyphal bodies showed that both BbMirA and BbMirB were distributed in EEs and LEs (Fig. 7B). Moreover, BbMirA-eGFP and BbMirB-RFP signals completely overlapped (Fig. S5A). These findings suggest that BbMirA and BbMirB likely co-localize at the plasma membrane, EEs, LEs, and vacuoles during fungal colonization of the host hemocoel. Vesicular trafficking within the eukaryotic endomembrane system involves vesicles that bud from donor organelles and fuse with target organelles (32). Soluble N-ethylmaleimide-sensitive factor attachment receptor (SNARE) proteins are essential for vesicle fusion events (33). A putative vesicle-SNARE protein gene, BBA_05832 (termed *Bbvsp1*), is located near the *BbMirB locus* and co-expressed in *B. bassiana* cells during host infection. To explore whether BbMirA and BbMirB trafficking is linked to Bbvsp1, we generated a strain expressing *Bbvsp1-mCherry* and *BbMirB-eGFP*. BbMirB was partially co-localized with Bbvsp1 (Fig. S5B), suggesting that BbMirB trafficking is associated with vesicle transport and membrane fusion. Dynamic imaging of *in vivo* hyphal bodies showed that both BbMirA-eGFP and BbMirB-eGFP co-localize and move with vesicles stained by FM4-64 (Fig. 7C; Videos S1 and S2), further supporting the hypothesis that BbMirA and BbMirB are trafficked in vesicles from the membrane via EEs and LEs to vacuoles.

### Disruption of *BbMirA* and *BbMirB* accelerated acidification of extra- and intra-cellular pH under iron-limited conditions

**Fig 8:**
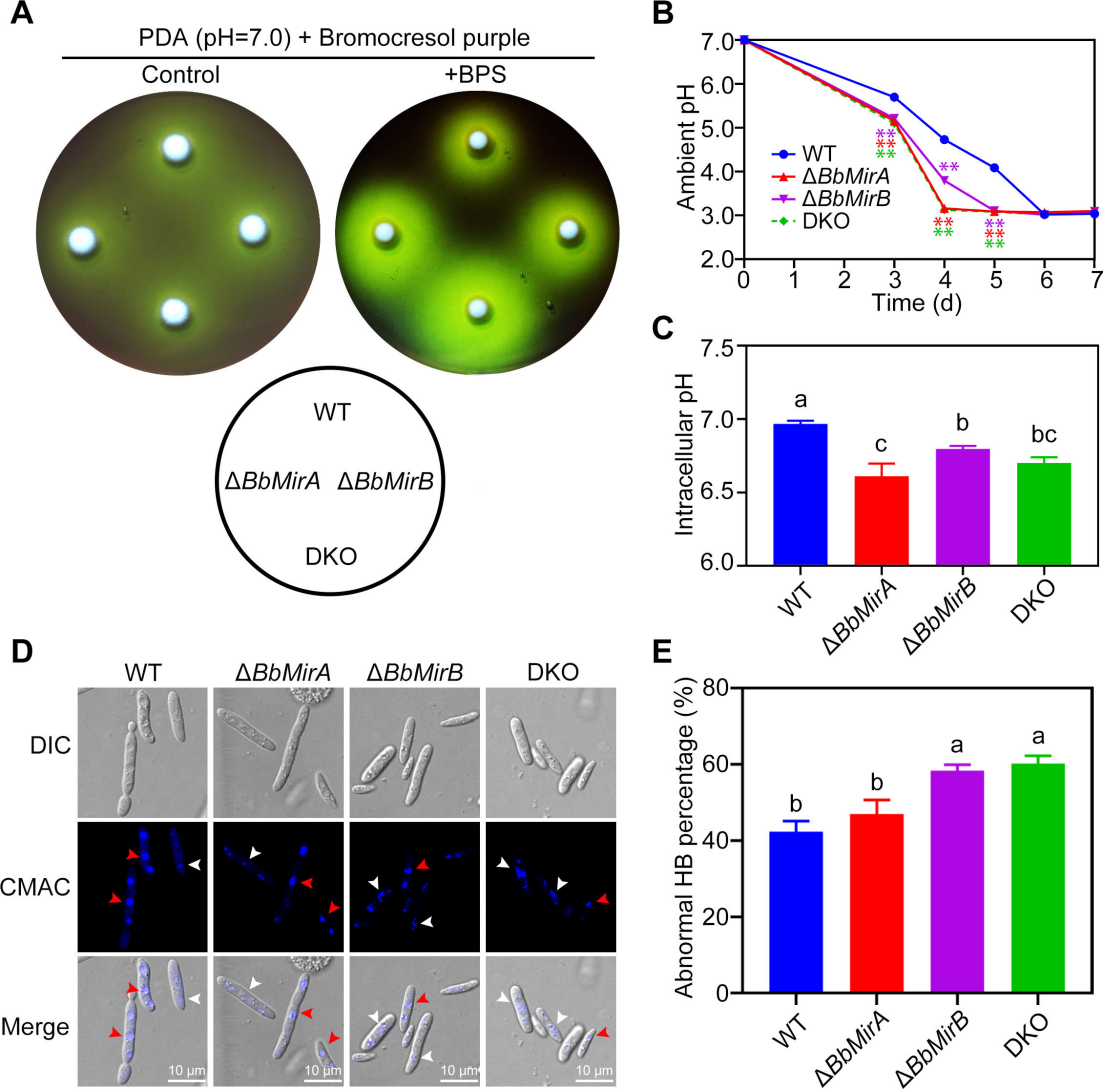
Extra- and intra-cellular acidification and vacuole morphology. (**A**) Acidification of fungal colony surroundings. WT, Δ*BbMirA*, and Δ*BbMirB* were applied on PDA with 200 µM BPS and 0.01% bromocresol purple (for indicating the ambient pH) and incubated at 26°C for 3 days. (**B**) Extracellular pH (mean ± SE, *n* = 3). The strains above were separately incubated in PDB with BPS on a shaker at 26°C, 180 rpm for 7 days, with pH values monitored from days 3 to 7. **P < 0.01, two-tailed Student’s *t*-test. (**C**) Intracellular pH (mean ± SE, *n* = 3). Fungal cells grown in PDB with BPS were harvested on day 7 by filtration, and the cytoplasm was extracted for pH determination using a pH meter. (**D**) Vacuoles of *in vivo* hyphal bodies. *In vivo* hyphal bodies were collected from infected larvae and stained using CMAC for vacuole observation. Red and white arrows indicate normal and abnormal (small and fragmented) vacuoles, respectively. (**E**) Ratio of *in vivo* hyphal bodies containing abnormal vacuoles (mean ± SE, *n* = 3). Lack of identical lowercase between groups presents a significant difference (*P* < 0.05, least significant difference [LSD] test). Bar = 10 µm.

A previous study in *Aspergillus nidulans* revealed that siderophore synthesis and uptake are regulated by the PacC-mediated pH-response system (34), indicating a connection between ambient pH and iron limitation adaptation. We investigated whether BbMirA and BbMirB affect ambient acidification by *B. bassiana*. Disruption of *BbMirA* and/or *BbMirB* did not affect fungal growth or acidification phenotype on PDA plates containing the pH indicator bromocresol purple (Fig. 8A). However, supplementation with BPS revealed increased acidification of the surrounding environment in Δ*BbMirA*, Δ*BbMirB*, and DKO mutants compared to WT, despite no observable differences in colony growth. Ambient pH measurements in PDB amended with BPS over 7 days indicated that Δ*BbMirA*, Δ*BbMirB*, and DKO mutants acidified their surrounding environment more rapidly than the WT (Fig. 8B). However, the pH values of the acidified broth converged by day 6, showing no significant differences. Subsequently, intracellular pH values were assessed by disrupting fungal cells on day 7. The mutants of Δ*BbMirA*, Δ*BbMirB*, and DKO exhibited significantly lower intracellular pH levels (6.61 ± 0.12, 6.80 ± 0.03, and 6.70 ± 0.06, respectively) compared to WT (6.97 ± 0.03), indicating interrupted pH homeostasis in the mutants (Fig. 8C).

Vacuoles are essential organelles for regulating intracellular pH homeostasis and extracellular acid-base balance (35). To assess the impact of *BbMirA* and *BbMirB* disruption on vacuolar morphology, we examined *in vivo* hyphal bodies using CMAC staining. Two types of vacuolar morphology were observed: smooth globular vacuoles (normal) and fragmented vacuoles (abnormal). The proportion of *in vivo* hyphal bodies containing abnormal vacuoles was significantly higher in Δ*BbMirB* (58.3% ± 2.2%) and DKO (60.2% ± 2.9%) compared to WT (42.3% ± 3.9%) and Δ*BbMirA* (47.0% ± 5.2%), respectively, with no noticeable difference between WT and Δ*BbMirA* (Fig. 8D and E). However, disruption of *BbMirA* or *BbMirB* did not affect the formation of EE or LE in *in vivo* hyphal bodies (Fig. S6).

## DISCUSSION

Invertebrates and vertebrates employ iron sequestration as a defense mechanism against pathogens (3, 36). SIA represents a critical mechanism by which pathogenic fungi overcome iron limitation (5). However, knowledge regarding SITs in fungal pathogens remains limited beyond *A. fumigatus* (20). Here, we characterized two SIT homologs, BbMirA and BbMirB, in *B. bassiana* Bb0062 that show high expression during host colonization and are co-expressed with a coprogen-type siderophore synthesis cluster (30). Deletion of *BbSidD* eliminated coprogen-type siderophore production, consistent with recent findings in *B. bassiana* ARSEF2860 (11), indicating a function of these transporters in siderophore trafficking.

Under iron limitation *in vitro*, *B. bassiana* Bb0062 produces primarily coprogen B, *trans*-fusarinine, and dimerumic acid with several derivatives, while in host hemolymph, a dimerumic acid derivative (C_22_H_40_N_4_O_10_) and *trans*-fusarinine predominate, proving *in vivo* production of siderophores in line with their previously shown role in virulence (11). Both Δ*BbMirA* and Δ*BbMirB* exhibited higher levels of coprogen B, dimerumic acid, and its derivative (C_22_H_38_N_4_O_9_) in iron-limited broth compared to wild type, indicating compromised siderophore uptake and/or augmented production due to iron deficiency. Notably, the content of a dimerumic acid derivative (C_22_H_40_N_4_O_10_) increased in Δ*BbMirB*- or DKO-infected hemolymph compared to WT- or Δ*BbMirA*-treated groups. However, DKO did not demonstrate significantly higher siderophore levels than single mutants in either iron-limited conditions or within host hemolymph. These findings suggest that both transporters facilitate coprogen-type siderophore uptake during iron-limited saprophytic growth, with BbMirB dominating the role *in vivo*. Bioassays confirmed that BbMirB, rather than BbMirA, is critical for *B. bassiana* virulence, as disruption of BbMirB alone or in combination with BbMirA impeded fungal proliferation within the host.

Nutritional immunity represents a significant defense mechanism in animals and insects against pathogen infection (3, 4). In *Drosophila melanogaster*, transferrin Tsf1 mediates this innate immunity against some invading pathogens (3), as well a tsf1 homolog (LOC113509694) shows significant upregulation in *B. bassiana*-infected *G. mellonella* larvae (37), indicating the involvement of host defense. However, the effectiveness of this defense varies by host-pathogen combination. For instance, *tsf1* knockdown in *D. melanogaster* does not affect survival against *B. bassiana* or *Candida albicans* (3), while similar knockdowns in *Plutella xylostella* and *Odontotermes formosanus* increase susceptibility to fungal pathogens (38, 39). Conversely, forager bees of *Apis mellifera* infected by *Nosema ceranae* display a significant reduction in total iron of fat body cells, and *tsf1*-knockdown increases tolerance to microspora (40). Our research demonstrates that BbMirB is more crucial than BbMirA in enabling *B. bassiana* to overcome host nutritional immunity, consistent with their relative contributions to virulence.

Transcriptional analysis demonstrated that larvae infected with Δ*BbMirB* exhibited significant upregulation of multiple immune-related genes in the fat body, while Δ*BbMirA* infection induced a more limited immune response. One possible explanation for the upregulation of insect immune defense genes in response to Δ*BbMirB* infection is that nutritional immunity promotes iron translocation from hemolymph to fat body, significantly reducing iron availability. This iron limitation affects cell wall remodeling in germinating conidia within insect hemolymph. A case in *C. albicans* has demonstrated that iron deficiency induces thickening of the outer cell wall, significantly increases the mannan content, while simultaneously increasing chitin and decreasing β-1,3-glucan contents in the inner wall (41). Cell wall surface profiles represent critical pathogen-associated molecular patterns essential for host recognition of pathogenic fungi and subsequent activation of immune-related gene expression (42, 43). This speculation requires further experimental verification. The differential pattern of host immune-related gene modulation, as well as evasion of immune encapsulation and proliferation *in vivo*, correlates directly with the observed virulence attenuation phenotypes, suggesting that BbMirB plays critical roles in fungal immune evasion mechanisms during infection.

In *A. fumigatus* and *F. graminearum*, SIT1 exclusively localizes to the plasma membrane (21, 44), while MirC is distributed on vacuole-like structures (45). *S. cerevisiae*’s SIT1 homolog and Arn1 are distributed on vacuoles and endosomes, with ENB1 confined to the plasma membrane (46). Microscopic fluorescence analysis of tagged proteins suggests that both BbMirA and BbMirB distribute on plasma membranes and co-localize to EEs/LEs and vacuoles of *in vivo* hyphal bodies in *B. bassiana*. We observed vesicles carrying BbMirA-eGFP/BbMirB-eGFP hybrid proteins moving from the plasma membrane to the vacuole, suggesting that these transporters may internalize extracellular siderophores by endocytosis and subsequently translocate them to the vacuole via vesicular transport. Alternatively, the localization of the two SITs in the different compartments reflects trafficking to the plasma membrane for substrate uptake and trafficking to the vacuole for degradation of the SITs. The consistent subcellular co-localization of BbMirA and BbMirB, as well as similar extracellular siderophores of mutants under iron starvation, indicated a possible cooperative role in transporting common substrates (such as coprogen B and dimerumic acid). Notably, fusion tags might affect protein localization and secretion, while the absence of specific antibodies against BbMirA and BbMirB precluded further confirmation of their subcellular distribution via immunoelectron microscopy.

*B. bassiana* acidifies its environment via oxalic and citric acid secretion (47), with enhanced efficacy under iron limitation. Fungi employ organic acids alongside siderophores for metal solubilization and chelation as adaptations to iron scarcity (48, 49), while siderophore synthesis and uptake are inhibited by ambient acidic pH (34). Therefore, the enhanced environmental acidification of Δ*BbMirA* and Δ*BbMirB* compared to the wild-type strain under iron limitation may indicate increased iron starvation of these mutants due to decreased siderophore-mediated iron uptake, i.e., the mutants try to compensate for the decreased siderophore-mediated iron uptake by environmental acidification. As SITs function as proton symporters (5), the lack of *BbMirA* and/or *BbMirB* may decrease the co-transport of protons into cells. Organic acid production is subject to feedback inhibition (50), preventing excessive environmental acidification, while the acidic pH inhibits siderophore uptake, attenuating the impact of *BbMirA* and *BbMirB* disruption on extracellular pH, thereby explaining the convergence of final extracellular pH values. Although both BbMirA and BbMirB localize to vacuoles—organelles crucial for pH homeostasis (35)—the correlation between intracellular pH reduction in mutants and vacuolar dysfunction remains unclear.

In conclusion, this study functionally characterized two SITs, BbMirA and BbMirB, which were highly expressed during the hemocoel-colonizing stage, specifically of *B. bassiana*. Our findings indicate that BbMirB is primarily responsible for the uptake of coprogen-type siderophores and plays a pivotal role in suppressing host immunity and promoting fungal proliferation *in vivo*. These results will advance our understanding of the roles of siderophores and their transporters during the hemocoel-colonizing stage.

## MATERIALS AND METHODS

### Strains and culture conditions

The WT strain *B. bassiana* Bb0062 (CGMCC 7.34) and its genetically modified mutants were grown on Czapek-Dox agar (Difco Laboratories, Detroit, MI) supplemented with 0.25% (wt/vol) peptone (CZP) at 26°C for 14 days for harvesting aerial conidia. Conidial suspensions were prepared in sterile 0.05% (vol/vol) Tween-80, and the concentration was adjusted to 10^7^ conidia/mL by a hemocytometer for counting. For liquid culture, conidia were incubated in 1/4 Sabouraud dextrose broth amended with 1% (wt/vol) yeast extract (SDY) at 26°C and 180 rpm for 60 hours on a rotatory shaker. *Escherichia coli* DH5α and *Agrobacterium tumefaciens* AGL-1 were employed for routine DNA manipulation and fungal transformation, respectively.

### Gene disruption, complementation, and fluorescence protein tagging

All primers used in this study are listed in Table S1. The sequence of the red fluorescent protein (RFP) gene was amplified from plasmid p1973 and cloned into pK2sur-GFP (51) at *Bam*HI/ *Eco*RV to replace e*GFP*, generating pK2sur-RFP. To probe expression profiles of target genes and subcellular localization of the proteins, the promoter regions or the full-length containing promoter and coding region (without stop code) were amplified from WT genomic DNA and inserted into pK2sur-GFP (51) and/or pK2sur-RFP at *Eco*RV, respectively.

To visualize EEs and LEs, the mCherry fluorescence protein was fused to the N-terminus of endogenous small GTPases BbRab5 and BbRab7 (52) and expressed in WT or mutants. Briefly, *mCherry* (without stop code) was amplified from the plasmid pBARGPE1-mCherry (Shenhe Biotech Co., Ltd, Shanghai, China) and cloned into pK2sur-Pb3 (53) at the *Bam*HI/*Eco*RV site to generate pK2sur-Pb3-mCherry. Subsequently, *BbRab5* and *BbRab7* coding sequences were amplified and inserted into pK2sur-Pb3-mCherry at *Eco*RV, respectively. For vesicle visualization, the amplified *Bbvsp1* (BBA_05832) was cloned into pK2sur-Pb3-mCherry as above.

Gene disruption was generated using homologous recombination, replacing the partial coding region of the target gene with a herbicide or sulfonylurea resistance gene (bar or sur) cassette, as previously described (54). Briefly, the 5′- and 3′-flanking sequences of the target gene were amplified and inserted into *Eco*RI and *Xba*I sites of pK2-bar (55) to create pK2bar-mirA^ko^ and pK2bar-mirB^ko^, or pK2bar-sidD^ko^, respectively. To construct the double-gene mutant, pK2sur-mirA^ko^ was also constructed as above based on the initial plasmid pK2sur (54) and was used to disrupt *BbMirA* in a *BbMirB*-disrupted mutant. To complement gene disruption mutants, full-length sequences, including the promoter, coding region, and terminator, were amplified from WT and cloned into the *Xba*I site of pK2sur (54) to yield pK2sur-mirA^com^ and pK2sur-mirB^com^, respectively.

To overexpress *BbMirA* or *BbMirB*, the cDNAs were amplified and inserted into the pK2sur-Pb3 (53) at the site of *Bam*HI, respectively, in which the target gene was under the control of the constitutive promoter Pb3 (56). All the resulting vectors were separately introduced into WT or corresponding mutants using *Agrobacterium*-mediated transformation.

### Gene expression analysis

Fungal cells of WT *in vitro* were prepared as previously described (57). *In vivo* blastospores, also known as *in vivo* hyphal bodies grown within host’s hemocoel, were prepared according to the previous report (53). Generally, last instar larvae of *G. mellonella* (a total of 30 larvae for each treatment) injected with conidial suspension (2 µL 10^7^ conidia/mL per larva) were bled at 48 hpi for *in vivo* hyphal body harvesting. BPS was used to establish an iron-depleted condition. Specifically, precultured fungal cells were inoculated into PDB or PDB containing 200 µM BPS and incubated for 6 hours.

A set of larvae (10 larvae in total for each treatment) was injected with conidial suspensions (2 µL 10^7^ conidia/mL per larva), and the fat bodies of the insect (three larvae in total for each treatment) were collected at 24 hpi for examining expression of genes associated with insect immune responses to fungal infection. Total RNA of all samples above was separately isolated using the TRIzol reagent (Invitrogen, USA), and single-stranded cDNA was synthesized using TRUE script RT Kit (Aidlab, China). RT-qPCR analysis was performed using the ChamQTM Universal SYBR qPCR Master Mix (Vazyme, China) on the CFX Connect Real-Time PCR Detection System (Bio-Rad, USA). The relative expression of each gene was normalized to the expression of the 18S rRNA gene (XM_008603008.1) in *B. bassiana* or β-actin (XM_026904349) in *G. mellonella*.

### Fluorescence microscopy

To visualize the expression profiles of *BbMirA* and *BbMirB*, fungal cells expressing *eGFP* under the control of the target gene promoter were prepared as “Gene expression analysis” (above). *G. mellonella* larvae were injected with 2 µL 10^7^ conidia/mL/larva of *BbMirA-eGFP*, *BbMirB-eGFP*, *mCherry-BbRab5*, and *mCherry-BbRab7*, respectively. At 48 hpi, the larvae were bled for the collection of *in vivo* hyphal bodies by centrifugation at 4°C and 6,000 rpm for 10 min, followed by washing twice with anticoagulant buffer (0.14 M sodium chloride, 0.1 M glucose, 26 mM citric acid, 30 mM sodium citrate, and 10 mM EDTA, pH 4.6). *In vivo* hyphal bodies were analyzed using a confocal laser scanning microscope (SP8, Leica), at the excision/emission wavelengths of 488/507 nm for eGFP, 580/610 nm for RFP, and 587/610 nm for mCherry, respectively. For visualizing the plasma membrane and vacuoles, the collected *in vivo* hyphal bodies were stained with FM4-64 or CellTracker Blue CMAC for 30 min, washed twice using anticoagulant buffer, and subsequently observed at the excision/emission wavelengths of 558/634 nm and 372/470 nm, respectively.

### Insect bioassays

To determine the effect of target gene disruption or overexpression on fungal virulence, insect bioassays were performed using the last instar larvae of *G. mellonella* by topical application and intra-hemocoel injection, respectively. Each treatment had three replicates (in a total of 90 larvae). For topical inoculation, 1 mL conidial suspension (2 × 10^7^ conidia/mL) was applied for each replicate using a spraying tower. For injection bioassays, 2 µL conidial suspension (10^5^ conidia/mL) per larva was injected into the insect hemocoel. The survival data were presented as Kaplan-Meier curves.

### Determination of fungal immune evasion and *in vivo* hyphal body proliferation

Each larva was injected with 2 µL 10^7^ conidia/mL of WT or mutants. To evaluate fungal immune evasion, treated larvae were bled at 24, 36, and 48 hpi, and both immune evasion (germ tubes penetrated through hemocyte encapsulation) and entire encapsulation events were observed and separately counted using microscopy, with the proportion of fungal immune evasion calculated. A total of nine larvae per treatment was examined, respectively. To determine concentrations of *in vivo* hyphal bodies within the insect hemocoel, 20 µL hemolymph derived from one treated larva was mixed with an equal volume of anticoagulant buffer, and *in vivo* hyphal bodies were recorded by direct count using a hemocytometer. A total of eight larvae per treatment were examined at 36, 48, and 60 hpi, respectively.

### Measurement of total iron concentration

To determine the total iron content in hemolymph or fat body, fungal conidia of each strain were dispersed into sterile 0.9% NaCl, adjusted to 10^7^ conidia/mL using a hemocytometer, and injected into hemocoel of insects (2 µL per larva, 30 larvae for each treatment), with the control larvae treated using sterile 0.9% NaCl only. Subsequently, 50 µL hemolymph per infected larva was collected, and fat bodies were dissected separately (seven insects in total for each treatment) at 24, 36, and 48 hpi. To eliminate *in vivo* hyphal bodies, collected hemolymph was centrifuged at 10,000 rpm (4°C, 8 min), while dissected fat bodies were washed three times with 0.9% NaCl. Total iron (Fe^2+^ and Fe^3+^) levels were measured using a commercial colorimetric kit (No. G1212W, Suzhou Geruisi-Bio) following the manufacturer’s protocol, with absorbance read at 562 nm. Protein concentrations of fat body extracts were quantified using the BCA Protein Assay Kit (No. GK5012, GENEray) to normalize the iron concentrations in fat bodies.

### Growth phenotypic, extra-, and intra-cellular acidification assays

To assess fungal growth under various stress conditions, 2 µL of conidial suspension (10^7^ conidia/mL) was inoculated onto plates of PDA or PDA supplemented with different stressors: 200 µM BPS, 2.5 mM FeCl_3_, 7.5 mM FeSO_4_, 1.3 M NaCl, 1.5 M KCl, 1.4 M sorbitol, 74 µM menadione, 3.17 mM H_2_O_2_, or 0.62 mM t-BHP. The plates were incubated at 26°C for 7 days. The values of relative growth inhibition for each stressor were calculated according to previously established methods (58) to quantify their inhibitory effects on fungal growth.

Bromocresol purple (0.01%) served as a pH indicator to evaluate colony-surrounding acidification. Equal conidial suspension aliquots (2 µL 10^7^ conidia/mL) were inoculated onto PDA or PDA plates amended with 200 µM BPS (adjusted to pH = 7.0 with 0.1 M NaOH) and incubated at 26°C for 3 days to monitor color changes. Additionally, strains above were separately cultured in PDB with BPS (10^5^ conidia/mL, pH 7) on a shaker at 26°C, 180 rpm for 7 days, with pH values monitored from days 3 to 7. Fungal cells were harvested by filtration on day 7, disrupted using a Tissue Cell-Destroyer, and the cytoplasmic pH was determined.

### Siderophore detection

For quantifying the secreted siderophores, precultured fungal cells were transferred into PDB containing 200 µM BPS and incubated for 48 hours at 26°C and 180 rpm on a rotatory shaker, followed by adding EDTA-Fe^3+^ (3 µM final concentration) and incubating for another 12 hours. Subsequently, the supernatant was collected by filtration for the siderophore measurement as follows. An aliquot of supernatant (3 mL) was mixed with an equal-volume Chrome azurol S (CAS) assay solution. After incubation in the dark for 30 min at room temperature, the absorbance of the 200 µL sample was measured at 630 nm on the Microplate reader (Varioskan LUX11005, Thermo), and the relative content of siderophore was calculated as previously described (59).

For detecting the siderophores in host hemolymph secreted by *in vivo* hyphal bodies of *B. bassiana*, *G. mellonella* larvae were separately injected with 2 µL of 10^7^ conidia/mL conidial suspension per insect, followed by bleeding at 48 hpi. The 500 µL hemolymph (collected from a total of 20 larvae) was mixed with an equal volume of anticoagulant buffer, followed by centrifugation at 4°C and 12,000 rpm for 10 min, and the supernatant was then collected. After drying using a freeze dryer and subsequently extracted with 300 µL methanol, the crude extracts were detected using an HPLC-ESI-TOF-MS 1290 Infinity II-6230 (Agilent, USA) with an EclipsePlus C18 column (21 mm × 150 mm, 1.8 µm). Analysis was performed using an H_2_O/acetonitrile gradient consisting of 10%–100% acetonitrile (0–8 min) and 100% acetonitrile (8–11 min), with a flow rate of 0.3 mL/min and a 10 µL sample injection.

### Data analysis

Statistical analyses were performed using SPSS 17.0 software with one-way analysis of variance followed by Fisher’s least significant difference or Dunnett’s T3 post-hoc test. The LT_50_ values in bioassays were calculated by probit analysis using SPSS. Specific statistical methods and parameters are detailed in the figure legends.

## SUPPLEMENTAL MATERIAL

Supplemental material mbio.01981-25-s0001.docx Tables S1–S3, Fig. S1–S6, and video legends.

Video S1 mbio.01981-25-s0002.mp4 BbMirA trafficking in *in vivo* hyphal bodies.

Video S2 mbio.01981-25-s0003.mp4 BbMirB trafficking in *in vivo* hyphal bodies.

ASM does not own the copyrights to Supplemental Material that may be linked to, or accessed through, an article. The authors have granted ASM a non-exclusive, world-wide license to publish the Supplemental Material files. Please contact the corresponding author directly for reuse.
