# Supplementary material for: The siderophore-iron transporter BbMirB is required for the fungal pathogen Beauveria bassiana to repress insect immunity and promote proliferation during colonization of hemocoel
Source: mBio. 2025 Aug 25;16(10):e01981-25. doi: 10.1128/mbio.01981-25 (PMC12506104; doi:10.1128/mbio.01981-25)
Supplement: Supplemental material — Tables S1–S3, Fig. S1–S6, and video legends. [file mbio.01981-25-s0001.docx]

**Supplementary Material**

**Chenhua Zhu, Qi Liu, Yuhan Chen, Fangfang Tian, Dekun Kong, Isidor Happacher, Hubertus Haas, Yongjun Zhang, Zhibing Luo**

# Supplementary Tables

Table S1 Primers used in this study

| Primer name | Sequence (5′ to 3′) | Remarks |
| --- | --- | --- |
| BbmirA-P1 | AGCTATGACCATGATTACGAATTCGCTTATTGGTACCTATGG | To construct pK2sur-mirA^ko^ |
| BbmirA-P2 | ATGTCATCTTCTGTCGACGAATTCCACGATAGATGTGTCGTC |  |
| BbmirA-P3 | TTAGAGGTAATCCTTCTTTCTAGATCGCTGCTTGTCTTATCC |  |
| BbmirA-P4 | GCATGCCTGCAGGTCGACTCTAGAGATAAAGTCACTATACAC |  |
| BbmirA-P5 | CGGATGAAAATGCAAAGACC | Δ*BbMirA* selection |
| BbmirA-P6 | GAGGTAATCCTTCTTTCTAGAATTACAGGCGTGTTGCCAG | To construct pK2sur-mirA^com^ |
| BbmirA-P7 | TGCCTGCAGGTCGACTCTAGACATCTATACTAGACT |  |
| BbmirA-P8 | TTTTAATCAATAACAGGATCCATGAAATCAGACGCTGC | To construct *BbMirA^OE^*/select DKO |
| BbmirA-P9 | ATTCCTGCAGCCCGGGGGATCCTTAATCGAGGGTCGATT |  |
| BbmirA-P10 | AAGCTTCTCGAGAGATCTGATACAGGCGTGTTGCCAG | To construct *P_BbmirA_-eGFP* and *BbMirA-eGFP* |
| BbmirA-P11 | CTCGCCCTTGCTCACCATGATATCGAGGGTCGATTTCGC |  |
| BbmirA-P12 | CTCGCCCTTGCTCACCATGATCGCTTCTTTTCTTGTTGT |  |
| BbmirB-P1 | AGCTATGACCATGATTACGAATTCCAGCCCGTGAAAGTGGAC | To construct pK2sur-mirB^ko^ |
| BbmirB-P2 | ATGTCATCTTCTGTCGACGAATTCGCTCCTTGGAACGATAGG |  |
| BbmirB-P3 | TTAGAGGTAATCCTTCTTTCTAGAGGGAAAAGTACTTTGCTC |  |
| BbmirB-P4 | GCATGCCTGCAGGTCGACTCTAGATACAGTGACTGATGATAG |  |
| BbmirB-P5 | AGACTGTGCGGTCCATGATG | To select Δ*BbMirB* |
| BbmirB-P6 | GAGGTAATCCTTCTTTCTAGATGGCTGAACGCTCTG | To construct pK2sur-mirB^com^ |
| BbmirB-P7 | TGCCTGCAGGTCGACTCTAGAGTACAAGGCTAGAAG |  |
| BbmirB-P8 | TTTTAATCAATAACAGGATCCATGCGCTTCTTTTCAAA | To construct *BbMirA^OE^*/select DKO |
| BbmirB-P9 | ATTCCTGCAGCCCGGGGGATCCTTAAACGACAGTTCCGT |  |
| BbmirB-P10 | AAGCTTCTCGAGAGATCTGATTGGCTGAACGCTCTGTGC | To construct *P_BbmirB_-eGFP* and *BbMirB-eGFP* |
| BbmirB-P11 | CTCGCCCTTGCTCACCATGATAACGACAGTTCCGTGAAC |  |
| BbmirB-P12 | CTCGCCCTTGCTCACCATGATGCGCATTGTCTTTTGGGG |  |
| BbmirB-P13 | TGCCTGCAGGTCGACTCTAGATGGCTGAACGCTCTGTGC | To construct *BbMirB-RFP* |
| BbmirB-P14 | CTCGGAGGAGGCCATGGATCCAACGACAGTTCCGTGAAC |  |
| RFP-F | TTTTAATCAATAACAGGATCCATGGCCTCCTCCGAGAAC | To construct pK2sur-RFP |
| RFP-R | GGTATCGATAAGCTTGATTAGATATCGGCGCCGGTGGAGTGGCG |  |
| mCherry-F | TTTTAATCAATAACAGGATCCATGGTGAGCAAGGGCGAG | To construct pK2sur-mCherry |
| mCherry-R | CGGTATCGATAAGCTTGATTAGATATCCTTGTACAGCTCGTCCAT |  |
| BbRab5-F | GGACGAGCTGTACAAGGATATGGCCTCCCGAGGACCC | To construct mCherry-BbRab5 |
| BbRab5-R | CGATAAGCTTGATTAGATTTAACAGCTGCAAGGGCC |  |
| BbRab7-F | GGACGAGCTGTACAAGGATATGTCTTCACGCAAGAAG | To construct mCherry-BbRab7 |
| BbRab7-R | TCGATAAGCTTGATTAGATTTAACAGGCACAGCCGTC |  |
| Bbvsp1-F | ATGCCTGCAGGTCGACTCTAGATCTCAATTATGGTTT | To construct *Bbvsp1-mCherry* |
| Bbvsp1-R | CGCCCTTGCTCACCATGGATCCTCGCAGGTAGTGCATCAC |  |
| q00636-F | GCTGCGACGTTTGAAGCACATC | RT-qPCR |
| q00636-R | TGGCTGATGAGCAGAACAAAGGC |  |
| q00825-F | AAGCAGCTGTCATACCCTGTGG |  |
| q00825-R | TCGCCATACGCCTTATCGATGG |  |
| q01825-F | TGCCTGCTCAACATGACCTACC |  |
| q01825-R | TCAAGAAGGGCTGCAAGTAGCTG |  |
| q03578-F | CTTTGCGCCCATCATTGGCTTC |  |
| q03578-R | TGATTGGCACGCCAGAGTAAGC |  |
| q05020-F | CAATGTTTCGCTGCACCTCG |  |
| q05020-R | TTCTTCAGCCTCTGCACCAC |  |
| q05816-F | AGCGAGTCGCCATTAACCAGAG |  |
| q05816-R | AGAAAGCGCCAGGAAAGCGATG |  |
| q06996-F | GGGGCGCTTCAAGGACATT |  |
| q06996-R | GGTCCGAGGGCTTCTTTGC |  |
| q06997-F | GCGCTACGAGCTCATCTTCT |  |
| q06997-R | TGGTAAACGTGGACAGGTCG |  |
| q07380-F | GTTTGGGCGCTTGGATTTGTCG |  |
| q07380-R | TGGCCTGCTTGATGCCAATGAC |  |
| q07619-F | AGTGTCATTGTCGGCACCTGTC |  |
| q07619-R | ACCGGCAATGATGAAGGGCTTG |  |
| q07649-F | TCGTACGGTCACGTCCAAAGAC |  |
| q07649-R | ACAAGCATGCGATGCAAATCGG |  |
| q08246-F | TGTGCAGTTTGGTAGCGTCT |  |
| q08246-R | AGTCTGCAAGATGACGGAGC |  |
| q09226-F | TTTCCGTCAGCCAAACCACAGC |  |
| q09226-R | ACCGAGCGACATGAAGATTTGGC |  |
| qBbmirA--F | TGGCTATTGATCGCAGCTTCCG |  |
| qBbmirA--R | ATGAGGTTCGGCACGCACAATG |  |
| qBbmirB--F | CGCGATTTGGACCGGTACTTTC |  |
| qBbmirB--R | TGGCGTAGATGGCATCAATGGG |  |
| 18S-F | ACGGGTAACGGAGGGTTAGG | Reference gene from *B. bassiana* |
| 18S-R | AGTACACGCGGTGAGGCGGA |  |
| *β*-actin F | ATCTGGCATCACACCTTCTACAACG | Reference gene from *G*. mellonella |
| *β*-actin R | GACATACATAGCCGGGGAGTTGAAG |  |

Table S2 Insect immune-related genes tested in this study.

| Gene name | Gene ID | Gene description |
| --- | --- | --- |
| *PGRP* | LOC113519541 | peptidoglycan recognition protein |
| *βgrp1* | LOC113513109 | β-1,3-glucan-binding protein-like |
| *Dorsal* | LOC113512577 | embryonic polarity protein dorsal |
| *Spätzle* | LOC113517666 | Spaetzle domain-containing protein 4 |
| *Gal* | LOC113523440 | gallerimycin-like |
| *Gal-P* | LOC113523425 | defensin-like |
| *Glo* | LOC113523269 | gloverin |
| *Prp1* | LOC113517304 | lebocin |
| *AP2* | LOC113519094 | anionic antimicrobial peptide 2-like |
| *Cec* | LOC113514266 | Cecropin-like |
| *Mor-A* | LOC113509609 | moricin-like peptide |
| *Mor-B* | LOC113509615 | moricin-like peptide B |

Table S3 Relative production of typical siderophores secreted by Δ*BbMirA*, Δ*BbMirB*, and Δ*BbSidD* as compared with WT^a^.

| Siderophore | Formula | Δ*BbMirA* | Δ*BbMirB* | Δ*BbSidD* |
| --- | --- | --- | --- | --- |
| Coprogen B | C_33_H_54_N_6_O_12_ | 7.36 ± 0.13 | 2.57 ± 0.17 | ND^b^ |
| Dimerumic acid | C_22_H_36_N_4_O_8_ | 4.09 ± 0.44 | 2.06 ± 0.52 | ND |
| Derivative of dimerumic acid | C_22_H_38_N_4_O_9_ | 6.39 ± 0.50 | 1.87 ± 0.19 | ND |
| Ferricrocin | C_28_H_47_N_9_O_13_ | 2.11 ± 0.02 | 1.69 ± 0.20 | 4.09 ± 0.36 |
| *trans*-Fusarinine | C_11_H_20_N_2_O_5_ | 0.92 ± 0.00 | 1.13 ± 0.03 | 0.90 ± 0.04 |
| Eleutherazine B | C_22_H_36_N_4_O_6_ | 1.09 ± 0.02 | 1.04 ± 0.07 | 1.11 ± 0.05 |

^a^: The siderophore level of the mutant was normalized to the production of WT based on their LC-MS peak areas. The data represent the mean of three replicates ± SE.

^b^: ND, not detected.

# Supplementary Figures

**
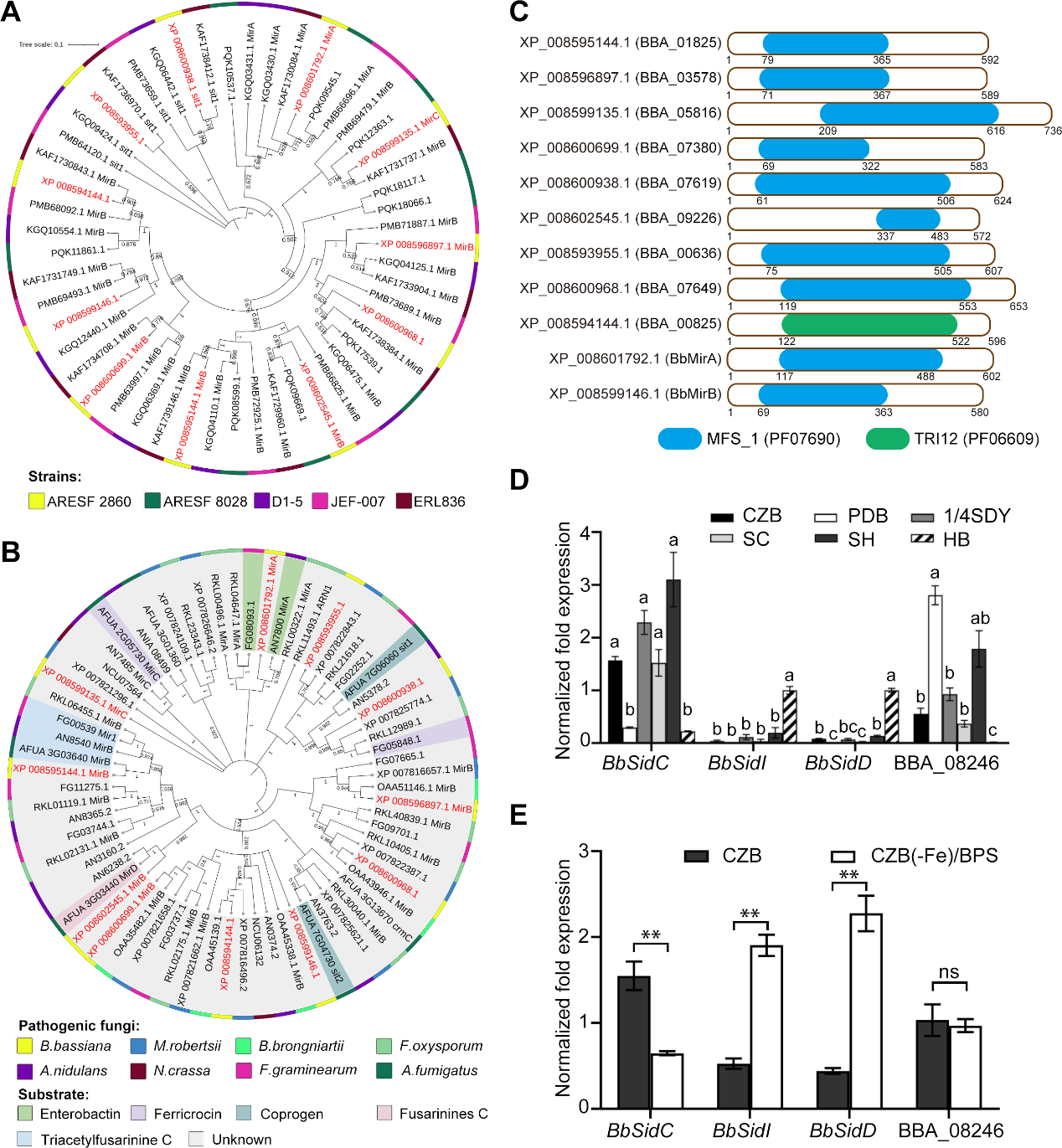
**

**FIG S1** Phylogenetic relationships of SITs and expression of core genes of siderophore biosynthesis. (A) Phylogenetic tree of SITs from several fungal species. (B) Phylogenetic tree of SITs from several Beauveria species. CLUSTAL_W, MEGA 7.0 and iTOLS (<https://itol.embl.de/>) programs were used to analyze SITs from several filamentous fungi. Species and the (putative) SITs are as follows, *B. bassiana* ARSEF 2860 (XP_008xxxxxx), *M. robertsii* ARSEF 23 (XP_0078xxxxx), *B. brongniartii* RCEF 3172 (OAAxxxxx), *F. oxysporum* (RKLxxxxx), *A. fumigatus* Af293 (AFUAxxxxxxx), *A. nidulans* FGSC A4 (ANxxxx), *F. graminearum* (FGxxxx), and *Neurospora crassa* (NCUxxxxx). Note: Here, the deduced amino acid sequence of BbMirA from *B. bassiana* Bb0062 contains an additional 116 amino acid fragment at the N-terminus as compared to the protein (BBA_08473) from ARESF 2860. (C) Conserved domains of putative SITs from *B. bassiana* (ARSEF 2860) were predicted using the program of InterPro (<https://www.ebi.ac.uk/interpro/>). (D and E) RT-qPCR analysis of core gene expressions in *B. bassiana* WT across various nutrient (D) or under iron-limited (E) conditions (mean ± SE, n=3). *BbSidC* (BBA_05020), *BbSidI* (BBA_06996) and *BbSidD* (BBA_06997), and BBA_08246 indicate the core genes within three clusters for siderophore biosynthesis, respectively. CZB, Czapek-Dox broth. PDB, Potato dextrose broth. 1/4SDY, diluted 1:4 of Sabouraud dextrose broth amended with 1% yeast extract. SC and SH, the basic salt broth supplemented with 1.67 g/ L silkworm cuticle or 5 mL/ L hemolymph. HB, *in vivo* hyphal bodies. PDB/BPS, PDB amended with 200 μM BPS. Different lowercase letters or ** indicate significant differences by LSD or Dunnett's T3 test at *P* < 0.05 (D) or two-tailed Student's *t*-test at *P* < 0.01 (E).

**
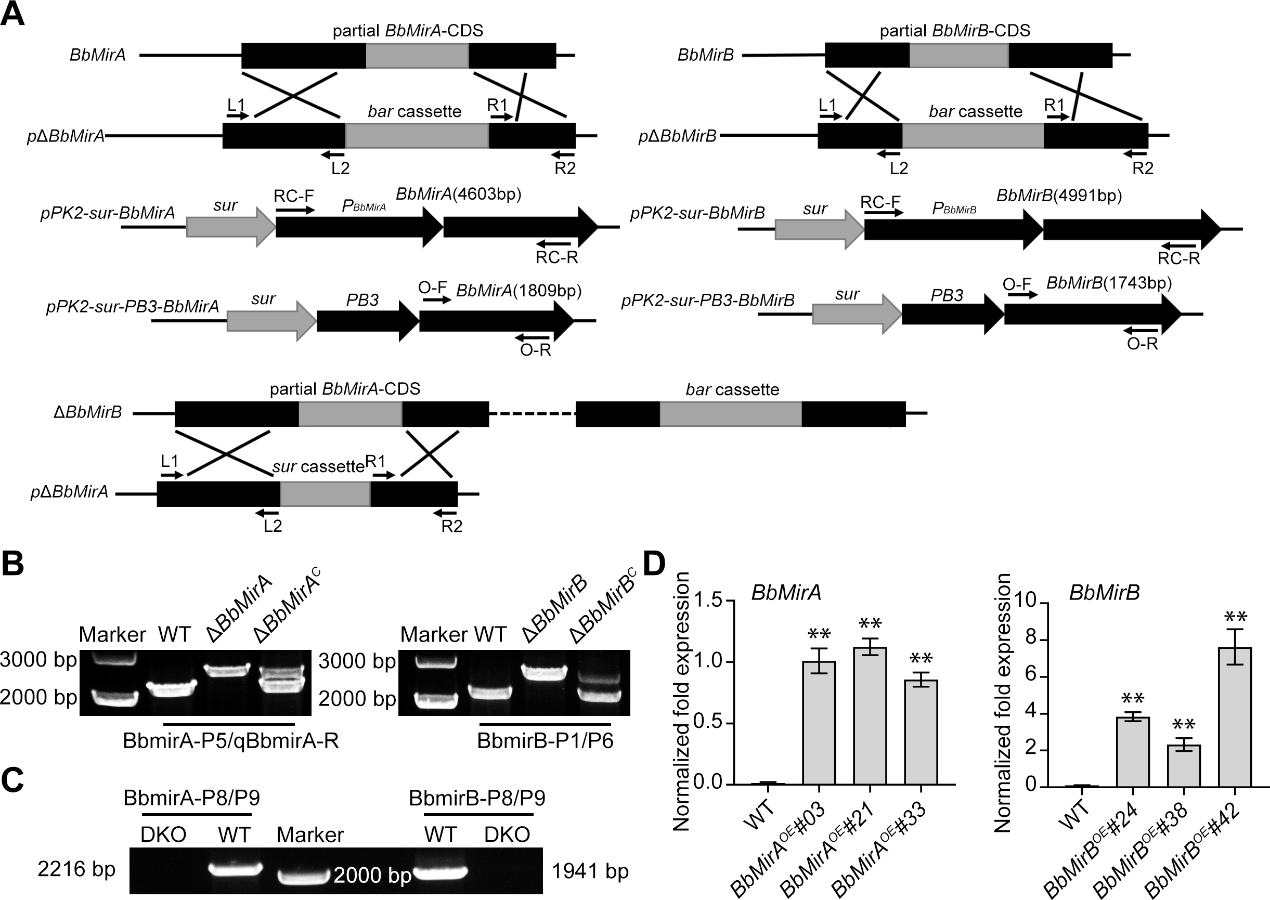
**

**FIG S2** Target gene disruption, complementation and overexpression. (A) Schematic diagram of disruption vector, genomic locus of target genes and homologous recombination events. L1/L2 and R1/R2 indicate primer positions for amplifying 5′- and 3′-flanking sequences of target gene. RC-F and RC-R represent the primer sites for amplification of *BbMirA* or *BbMirB* full length including promoter, terminator and coding sequence. O-F and O-R show the primer locations for cloning target gene ORF. (B) PCR confirmation of *BbMirA*- or *BbMirB*-disrupted and complemented events. BbmirA-P5/qBbmirA-R and BbmirB-P1/P6 are the primer pairs for PCR confirmation. (C) PCR confirmation of double-knockout events. BbmirA-P8/P9 and BbmirB-P8/P9 are the primer pairs for PCR confirmation. (D) Screening of *BbMirA* or *BbMirB* overexpressing transformants by RT-qPCR (mean ± SE, n=3). ** represents significant difference between overexpression strain and WT (two-tailed Student's *t*-test, *P* < 0.01).

**
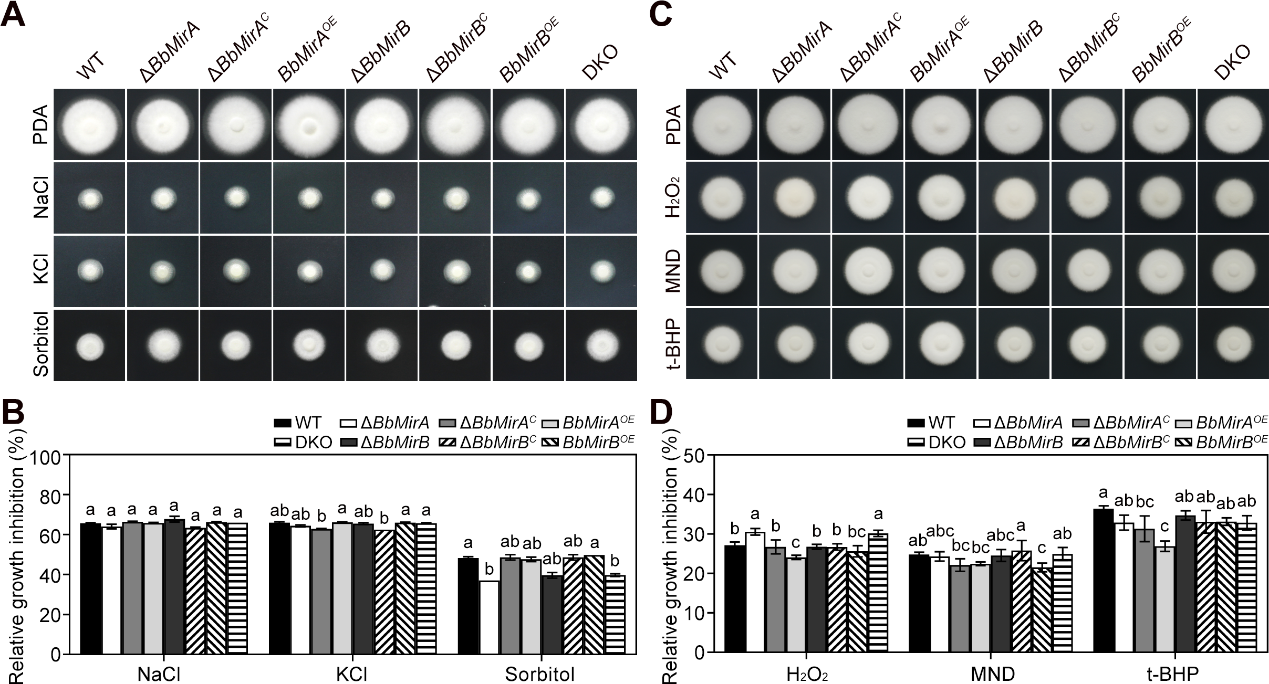
**

**FIG S3** Growth phenotypes under multiple stress conditions. (A) Fungal growth under hyperosmotic stress conditions. (B) Relative growth inhibition values of hyperosmotic stressors. (C) Fungal growth under oxidative stress conditions. (D) Relative growth inhibition values of oxidants. Equivalent conidial suspensions (2-μL 10^7^ conidia/mL) were applied on PDA plates, either non-amended or supplemented with hyperosmotic agents (1.3 M NaCl, 1.5 M KCl, or 1.4 M sorbitol), or oxidative stressors (3.17 mM H_2_O_2_, 74 μM MND, or 0.62 mM t-BHP). All cultures were maintained at 26°C for seven days. Relative growth inhibtion values were calculated according to the colony diameters (mean ± SE, n = 3). Lack of identical lowercase letters between groups indicates statistically significant differences (*P* < 0.05, Dunnett’s T3 or LSD test).

**
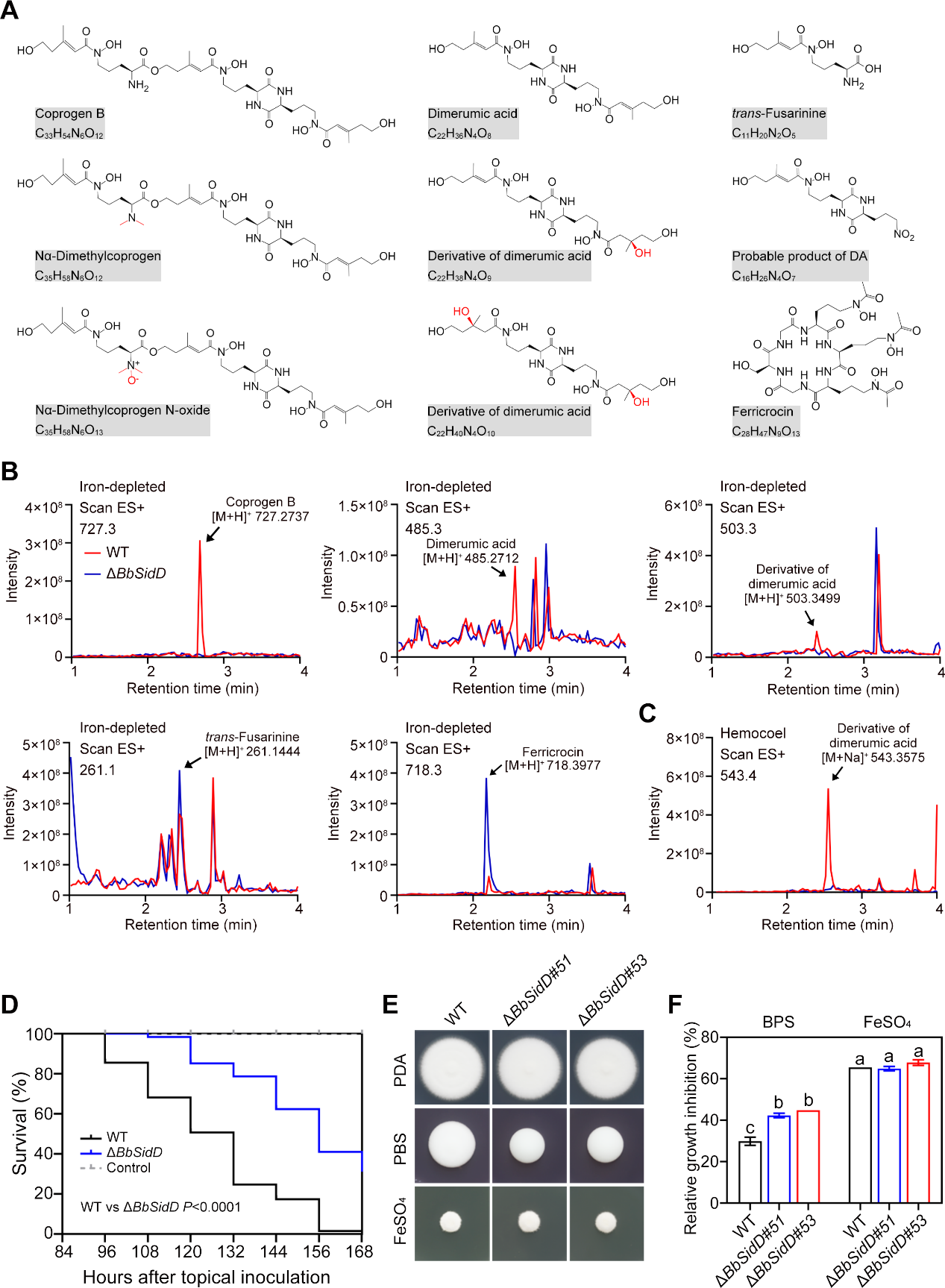
FIG S4** Disruption of *BbSidD* abrogated coprogen-type siderophore synthesis, impaired the virulence and iron hemostasis. (A) The molecular structures of typical siderophores secreted by *B. bassiana* Bb0062 WT under iron-limited condition and within host hemocoel. (B and C) Representative typical siderophores produced by WT and Δ*BbSidD* under iron-limited condition (B) or within host hemocoel (C). (D) Insect bioassays. The last instar larvae of *G. mellonella* were inoculated with WT and Δ*BbSidD* *via* spraying with 1-mL of 2 × 10^7^ conidia/mL (per replicate) and insect survival was then recorded. Statistical significance was assessed *via* a Log-rank test for pairwise survival curve comparisons. (E) Fungal growth under iron-limited or iron-replete conditions. (F) Relative growth inhibition values of stressors. Conidial suspension (2-μL of 10^7^ conidia/mL) was dropped on PDA plates, either non-amended or supplemented with BPS (200 μM) or FeSO_4_ (7.5 mM). All cultures were incubated at 26 °C for seven days. Relative growth inhibition values were calculated according to the colony diameters (mean ± SE, n = 3). Lack of identical lowercase letters between groups indicate statistically significant differences (*P* < 0.05, LSD test).


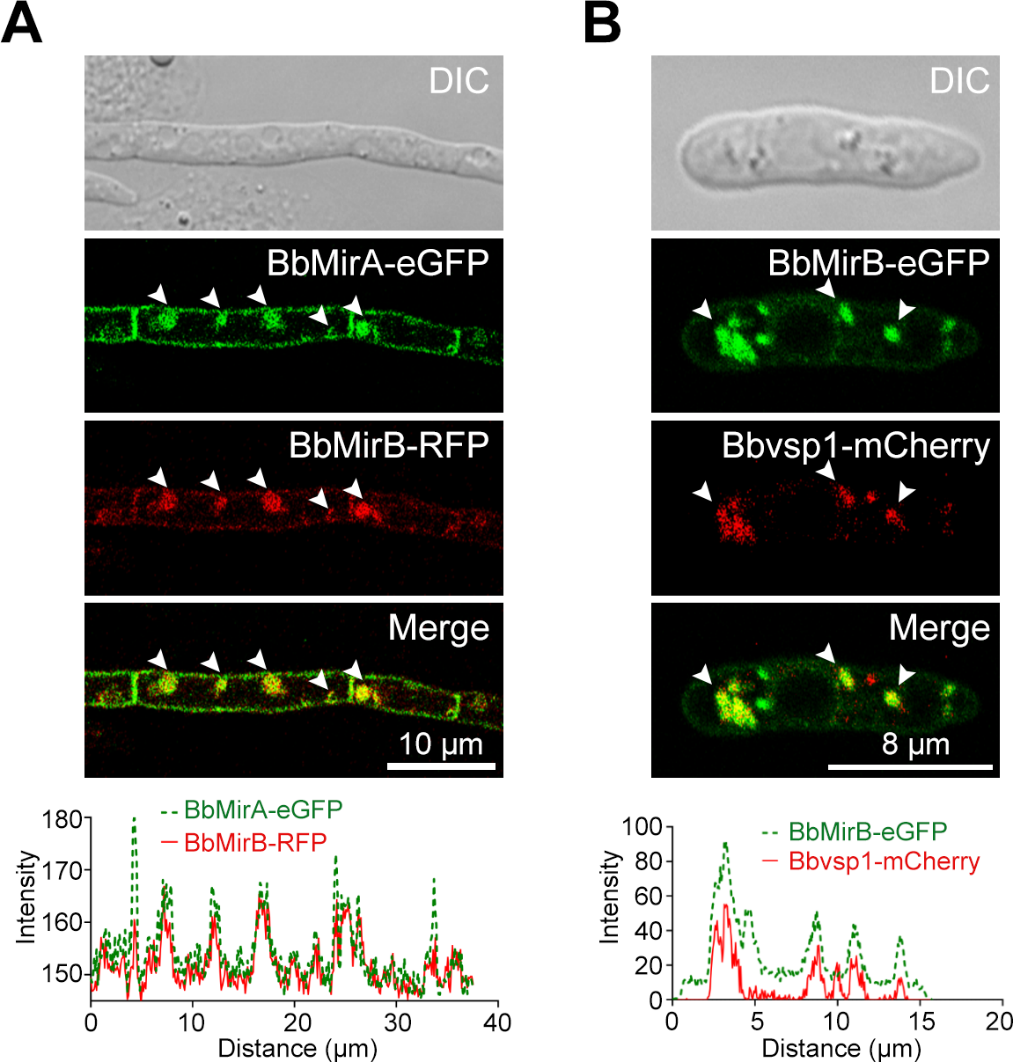


**FIG S5** Subcellular co-localization of BbMirA, BbMirB, and Bbvsp1. (A) Subcellular co-localization of BbMirA and BbMirB. (B) Subcellular co-localization of BbMirB and Bbvsp1 partially. *In vivo* hyphal bodies expressing BbMirA-eGFP/ BbMirB-RFP (A) or BbMirB-eGFP / Bbvsp1-mCherry (B) were isolated from infected larvae at 48 hpi and observed using confocal microscopy. White arrows indicate fluorescence signals overlapped (up panel). Co-localization of the proteins was further evaluated by linescan graph analysis using Image J (horizontal axis indicates the distance) (down panel).


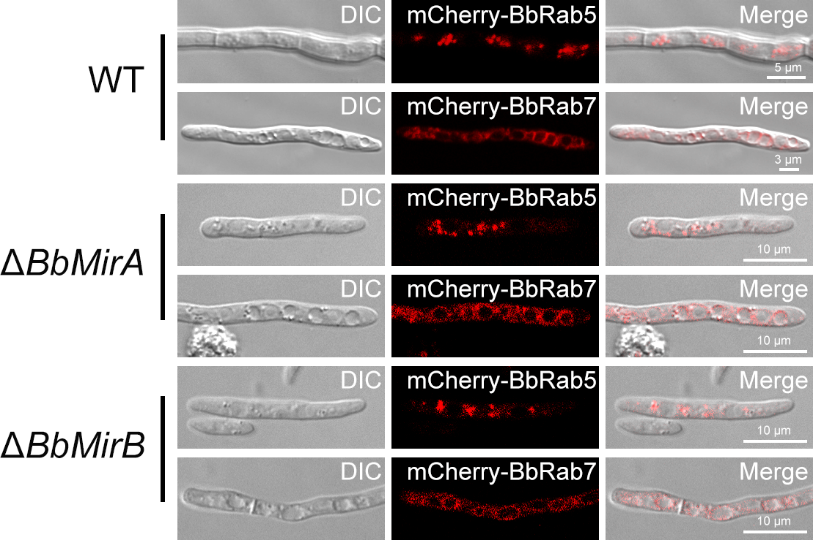


**FIG S6** EEs and LEs of *in vivo* hyphal bodies. *G. mellonella* larvae were subjected to intra-hemocoel injection with wild-type, Δ*BbMirA*, or Δ*BbMirB*, each expressing fluorescent endosomal markers (mCherry-BbRab5 or mCherry-BbRab7). *In vivo* hyphal bodies were subsequently harvested from infected larvae at 48 hpi and visualized using confocal laser scanning microscopy.

# Supplementary videos

Video S1. BbMirA trafficking in *in vivo* hyphal bodies*. In vivo* blastospores were isolated from larvae infected by ΔBbMirA carrying BbMirA-eGFP at 48 hpi, which were then stained by FM4-64 for 30 min, followed by DIC and fluorescent microscopy.

Video S2. BbMirB trafficking in *in vivo* hyphal bodies. *In vivo* blastospores were isolated from larvae infected by ΔBbMirB expressing BbMirB-eGFP at 48 hpi, which were then stained by FM4-64 for 30 min, followed by DIC and fluorescent microscopy.
